# Systemic distribution of medullary bone in the avian skeleton: ground truthing criteria for the identification of reproductive tissues in extinct Avemetatarsalia

**DOI:** 10.1186/s12862-019-1402-7

**Published:** 2019-03-07

**Authors:** Aurore Canoville, Mary H. Schweitzer, Lindsay E. Zanno

**Affiliations:** 1 Paleontology, North Carolina Museum of Natural Sciences, Raleigh, NC USA; 2 Department of Biological Sciences, North Carolina State University, Raleigh, NC USA

**Keywords:** Neornithes, Medullary bone skeletal distribution, Skeletal pneumaticity, Laying cycle, Micro-computed tomography, Chemical staining, Cranial medullary bone, Bone marrow, Avian ecology

## Abstract

**Background:**

Medullary bone (MB) is an estrogen-dependent, sex-specific tissue produced by female birds during lay and inferred to be present in extinct avemetatarsalians (bird-line archosaurs). Although preliminary studies suggest that MB can be deposited within most skeletal elements, these are restricted to commercial layers or hormonally treated male pigeons, which are poor analogues for wild birds. By contrast, studies in wild bird species noted the presence of MB almost exclusively within limb bones, spurring the misconception that MB deposition is largely restricted to these regions. These disparate claims have cast doubt on the nature of MB-like tissues observed in some extinct avemetatarsalians because of their “unusual” anatomical locations. Furthermore, previous work reported that MB deposition is related to blood supply and pneumatization patterns, yet these hypotheses have not been tested widely in birds.

To document the skeletal distribution of MB across Neornithes, reassess previous hypotheses pertaining to its deposition/distribution patterns, and refine the set of criteria by which to evaluate the nature of purported MB tissue in extinct avemetatarsalians, we CT-scanned skeletons of 40 female birds (38 species) that died during the egg-laying cycle, recorded presence or absence of MB in 19 skeletal regions, and assessed pneumatization of stylopods. Selected elements were destructively analyzed to ascertain the chemical and histological nature of observed endosteal bone tissues in contentious skeletal regions.

**Results:**

Although its skeletal distribution varies interspecifically, we find MB to be a systemic tissue that can be deposited within virtually all skeletal regions, including cranial elements. We also provide evidence that the deposition of MB is dictated by skeletal distribution patterns of both pneumaticity and bone marrow; two factors linked to ecology (body size, foraging). Hence, skeletal distribution of MB can be extensive in small-bodied and diving birds, but more restricted in large-bodied species or efficient flyers.

**Conclusions:**

Previously outlined anatomical locations of purported MB in extinct taxa are invalid criticisms against their potential reproductive nature. Moreover, the proposed homology of lung tissues between birds and some extinct avemetatarsalians permit us to derive a series of location-based predictions that can be used to critically evaluate MB-like tissues in fossil specimens.

**Electronic supplementary material:**

The online version of this article (10.1186/s12862-019-1402-7) contains supplementary material, which is available to authorized users.

## Background

Medullary bone (MB) is a sex-specific, estrogen-dependent tissue unique to female birds among extant amniotes [1], produced during the egg-laying cycle [2]. Together with dietary intake, MB is a key source of calcium necessary for the formation of avian eggshell [3–6]. This unique adaptation was first observed by Foote [7, 8] in the femora of a passeriform and pelecaniform, and later rediscovered by Kyes and Potter [9] in the hind limbs of female columbiforms; however, the majority of subsequent MB research centered around species and questions pertinent to the commercial poultry industry, which are of limited utility for exploring evolutionary and ecological trends in avian reproductive biology.

The unique microstructure and chemistry characterizing MB arise from its role in rapid mobilization of calcium for mineralization of eggshell [10]. Indeed, MB is a highly vascularized, mostly woven, endosteally-derived tissue [2, 11], with a unique molecular composition [10, 12–15]. These microstructural and chemical criteria (discussed in, for example, [16–19]) have been used to identify MB-like tissues in extinct non-avian clades diverging directly from the avian lineage (Avemetatarsalia), with pronounced implications for the origin and evolution of this tissue and the specialized reproductive strategy of Avialae.

Schweitzer et al. [20] were the first to report the presence of MB-like tissues in an extinct species of avemetatarsalian, specifically within the hind limbs of *Tyrannosaurus rex* (MOR 1125). This study generated interest within the paleontological community because, except in rare cases of exceptionally well-preserved specimens (e.g., a gravid female of an oviraptorosaur NMNS-VPDINO-2002-0901 found with ready-to-be-laid eggs in its body cavity, [21]), indicators of sex in extinct animals are usually lost during fossilization. The unambiguous identification of MB in extinct avemetatarsalians would provide an objective means to recognize sexually mature females, giving access to key data for understanding a whole host of ecological and evolutionary questions that would otherwise remain inaccessible. In the years since Schweitzer et al. [20] first reported MB in *T. rex*, MB-like tissues have been identified in a variety of extinct avemetatarsalians, including pterosaurs [22, 23], a diverse array of non-avian dinosaurs [17, 24–28], and extinct birds [29–33]. Some of these identifications were later questioned and hypothesized to be pathological as opposed to medullary bone tissues. The purported nature and reproductive function of some of these endosteal tissues were also challenged based, in part, on their unusual anatomical locations (such as within the mandibular symphyses of pterosaur specimens [19, 23]; see also [27, 33, 34]).

To date, the skeletal distribution of MB in extant birds has been poorly documented. Taylor and Moore [35] reported that, although only present in small amounts in the skull, humerus, and autopod, MB could be observed in most skeletal elements of laying pullets, and a similar distribution was noted in estrogen-treated male pigeons [36]. However, because these studies are restricted to commercial layers or hormonally treated birds, they are poor analogues for wild bird species, fail to capture potential variation associated with differences in avian physiology, ecology, and reproductive biology, and are not representative of Neornithes phylogenetic diversity. Other studies, which addressed MB structure, composition, and metabolism in wild and domesticated birds, focused almost exclusively on long limb bones [11, 37–41], spurring the common misconception that MB deposition is concentrated within limb bones [2, 42]. There is, therefore, a need to better document MB skeletal distribution across Neornithes, to provide a baseline for identifying purported MB-like tissues in extinct organisms [19, 41].

To begin to address the lack of data on both the phylogenetic and skeletal distribution of MB in Aves, Werning [43] investigated the occurrence of MB across a large sample of living birds, primarily relying on a candling technique, and briefly discussed the interspecific variation of its skeletal distribution. While providing an initial baseline, this study was limited in focus, relying primarily on long limb bones and only considering select girdle elements (coracoid, scapula, pelvis) in a subsample of species ([43]: appendix 2). Werning [43] found: 1) that MB was often present in the femora, ulnae, and proximal tibiotarsi of female laying birds, but rarely observed in tarsometatarsi and distal tibiotarsi; 2) MB was found in large quantity in the humeri of several species, contrary to previous predictions [44]; 3) MB was absent in the femora of Falconidae and some Accipitriformes; 4) these candling data suggested a less extensive skeletal distribution of MB in wild birds than in captive or hormonally treated specimens.

Several studies have proposed hypotheses to explain the skeletal distribution pattern of MB in extant birds. Previous works report that the amount of MB deposited in avian hind limbs follows a decreasing proximo-distal gradient from stylopod (femur) to autopod (tarsometatarsus and pedal phalanges), hypothesized to result from 1) a decreasing blood supply (and thus a decreasing amount of estrogen) distally throughout the limb [38]; and/or 2) differential receptivity of skeletal elements to hormones [45, 46]. A link between blood supply and MB metabolism was supported by Taylor and Moore [47] who observed that MB only forms in skeletal elements containing hematopoietic tissue. However, MB is expected to be scarce or absent in pneumatized elements such as the humerus [44], because these are commonly invaded by diverticula from the clavicular air sac [38, 48]. Although Werning’s results [43] supported the relationship between MB deposition and presence of red bone marrow, an inverse relationship between the skeletal distribution of MB and pneumatization was not upheld.

**Fig. 1:**
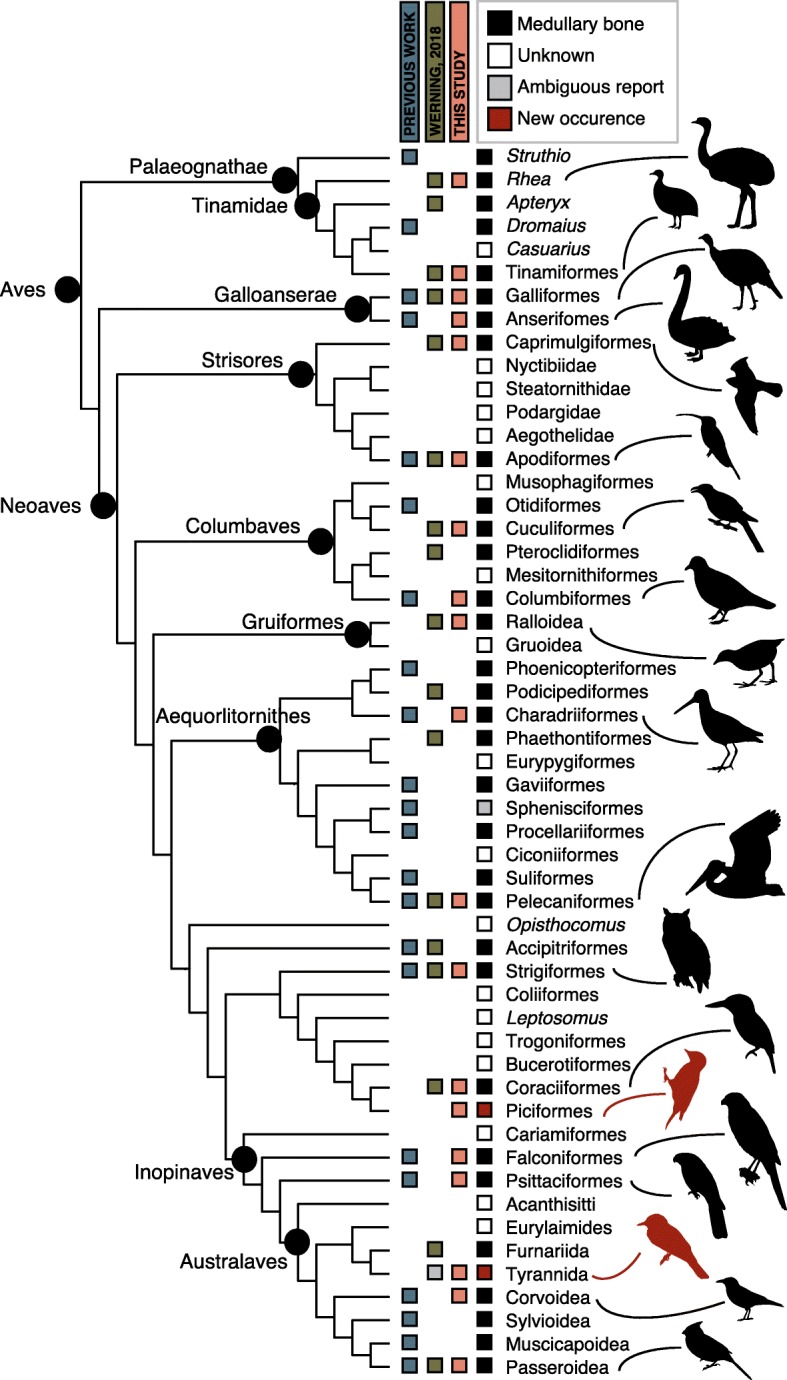
Known phylogenetic distribution of MB in Neornithes. The phylogenetic relationships of extant bird groups follow Prum et al. [52]. Superimposed upon this phylogeny is the documented presence of MB, based on data gathered by Werning [43] and the current study

Here we explore the skeletal distribution of MB in a comprehensive sample of living birds (Fig. 1), spanning avian phylogeny and including a diverse array of ecologies. We document distribution of MB across the complete avian skeleton using non-invasive micro computed-tomography (μCT), independently confirm MB tissues in historically controversial skeletal regions via histochemistry, and reassess previous hypotheses pertaining to its distribution patterns. Our results refine the set of criteria by which to evaluate purported MB tissue in fossil avemetatarsalians.

## Results

### Intraspecific variability

**Fig. 2:**
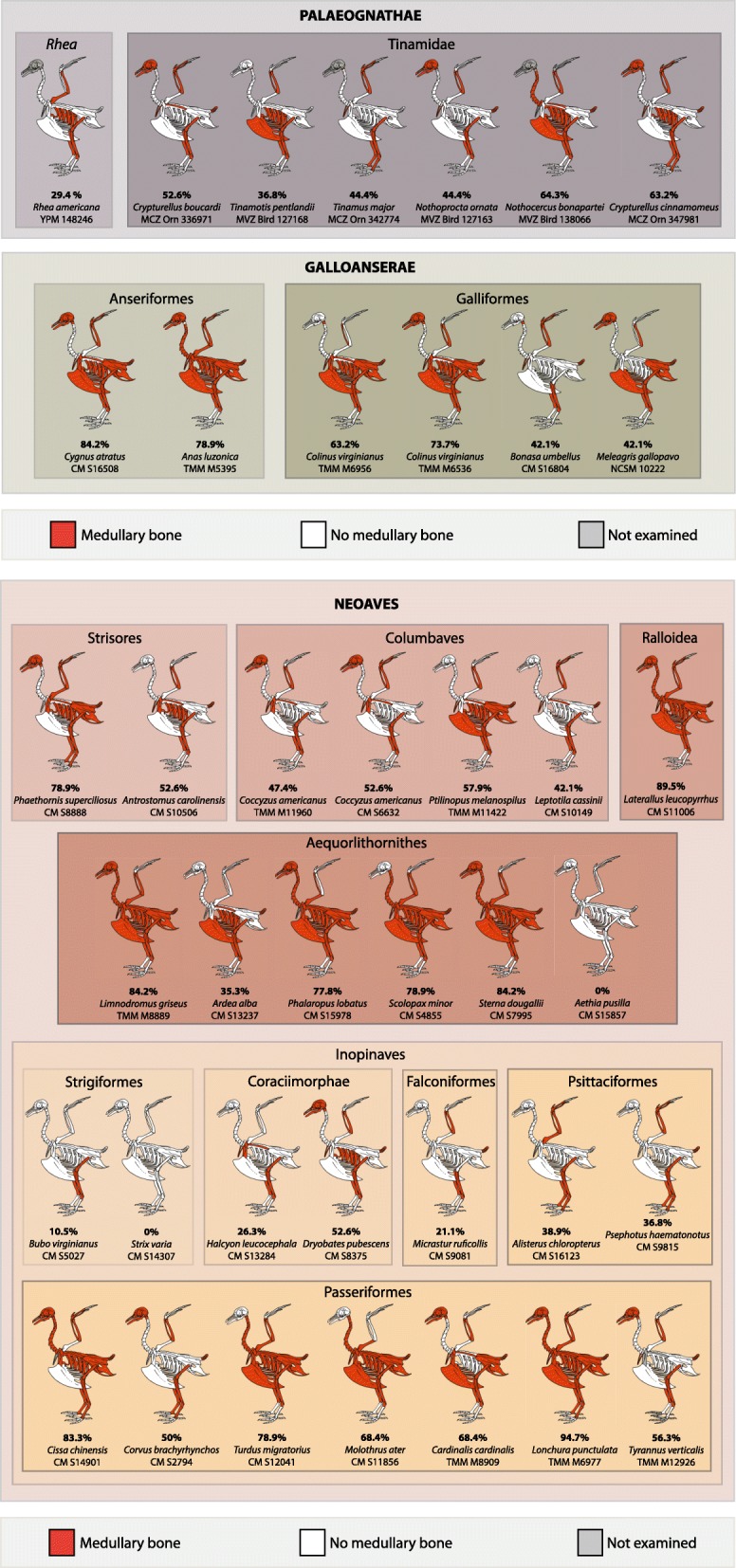
Extent and pattern of MB skeletal distribution in all 40 specimens sampled. The extent of MB skeletal distribution is expressed in % of skeletal regions containing MB. The specimens are classified following the phylogeny of Prum et al. [52]. The skeletal scheme used here is representative of the general bird skeleton depicting all considered elements, regardless of the species

Multiple reproductively active females of the same species were rare in our sample (*n* = 2), but include the galliform *Colinus virginianus* (northern bobwhite, TMM-M6536 and TMM-M6956) and the cuculid *Coccyzus americanus* (yellow-billed cuckoo, TMM-M11960 and CM-S6632). Specific information about the reproductive status of northern bobwhite specimens were not available; however, both exhibit a similar distribution of MB throughout the skeleton (Additional file 1: Table S1; Fig. 2). Potential variability was observed in the skull and ulna. TMM-M6536 definitively preserved small amounts of MB in these regions, whereas the presence of MB in the skull and ulna of TMM-M6956 was ambiguous. Limited variability was observed within the two specimens of the yellow-billed cuckoo (Additional file 1: Table S1; Fig. 2). Within the head and neck, both specimens deposited MB in the skull and atlas, but not within the axis. Similarly, both specimens deposited MB within the coracoids, scapulae and furcula, yet not the sternum. The only difference observed is the presence of MB within the carpometacarpus of CM-S6632, and its absence in TMM-M11960. Although limited, these results generally suggest little intraspecific variability in MB distribution within different regions of the skeleton.

### Interspecific variability

Only two skeletons in our sample lack a clear indication of MB (Additional file 1: Table S1)—the least auklet *Aethia pusilla* (CM-S15857) and a barred owl *Strix varia* (CM-S14307). The least auklet perished at the end of the laying cycle bearing a shelled egg within the oviduct. These birds lay only a single egg per clutch [49]; therefore, the majority of MB tissue would have been resorbed at the time of death, and the absence of MB in this specimen is unsurprising. The barred owl also died during the laying cycle, with an egg in the oviduct. We can thus assume that, as in the least auklet, MB of this specimen had already been resorbed at the time of death. Because the absence of MB in these specimens is likely related to the period of sampling (at the termination of the lay-cycle), we excluded them from calculations of the prevalence of MB.

The extent and pattern of MB skeletal distribution varies greatly between the species considered (Additional file 1: Table S1; Fig. 2). Some birds contain MB in up to 95% of the skeletal regions considered. The scaly-breasted munia (*Lonchura punctulate*, TMM-M6977) died egg-bound (a pathological condition resulting in retention of a fully formed shelled-egg within the oviduct), yet this bird possesses small amounts of MB in most skeletal regions (Fig. 2d), with the exception of the pedal phalanges.

In contrast, some specimens, estimated to be at the beginning or middle of the laying cycle, exhibit MB in ≤20% of the skeletal regions examined. The great horned owl *Bubo virginianus* (CM-S5027) has MB only within its femora and tibiotarsi (10.5% of its skeleton); the barred forest falcon *Micrastur ruficollis* (CM-S9081) exhibits MB within its radii, ulnae, tibiotarsi, and small amounts in its caudal vertebrae/pygostyle (21.1% of skeletal regions).

We observe no direct relationship between the quantity and skeletal distribution of MB and body-size or clutch size within our sample (Additional file 1: Table S1). Large-bodied species that lay large clutches of 5–10 eggs, such as *Rhea americana* YPM-148246 (weight of adult female = 20–25 kg; [49]) exhibit MB in only 29.4% of the skeleton (mostly in limb bones). By contrast, the second largest species in our sample (the black swan *Cygnus atratus,* CM-S16508; weight of adult female = 3.7–7.2 kg; 4 to 6 eggs per clutch; [49]), and medium-sized birds such as the roseate tern (*Sterna dougallii*, CM-S7995; 90–125 g; average 1–2 eggs per clutch) deposit MB in 84.2% of their skeletons. The smallest specimen in our sample, the hummingbird *Phaethornis superciliosus* (CM-S8888), has an average clutch size of two eggs for an adult female weighing 2 to 4 g [49], and 78.9% of its skeletal elements contain MB.

We also find no obvious difference in the pattern and extent of MB skeletal distribution between specimens that were caught in the wild and those that lived and died in captivity (Additional file 1: Table S1). For instance, *Limnodromus griseus* (TMM-M8889) and *Sterna dougallii* (CM-S7995) were caught in the wild, and yet exhibit an extensive distribution of MB in more than 84% of the skeletal regions considered. Moreover, the wild-caught *Limnodromus griseus* (TMM-M8889) and the captive *Laterallus leucopyrrhus* (CM-S11006) share a similar distribution of MB.

### Differences in the prevalence of MB in the various skeletal regions

**Fig. 3:**
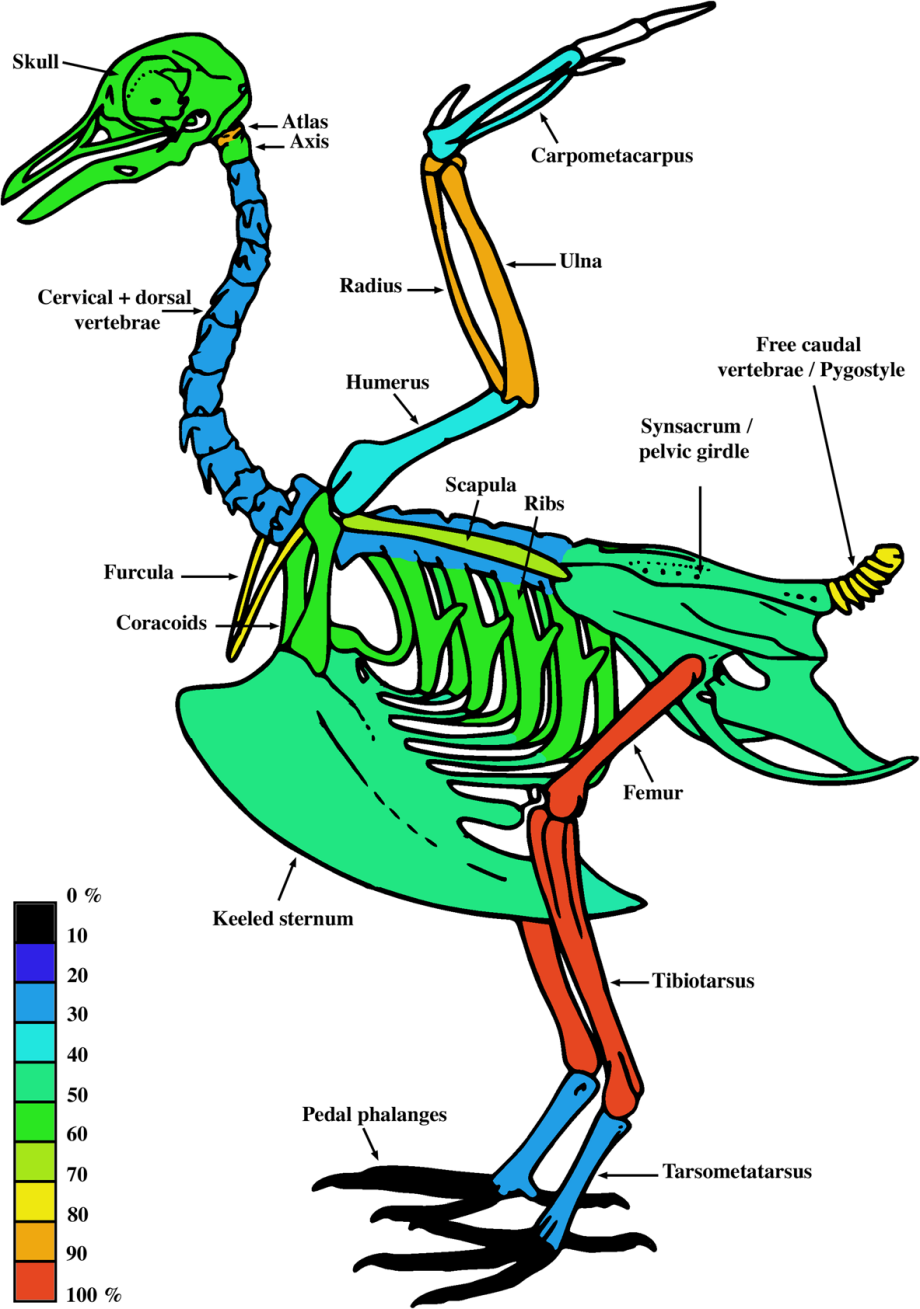
Overall distribution and prevalence of MB in the different regions of the avian skeleton. These results are based on our dataset comprising 38 specimens representative of extant bird diversity. The color-coding refers to the proportion of individuals in our sample (expressed in %) that presented MB in a given skeletal element. For example, 30 to 40% of the studied specimens presented MB in the humerus. The presence of MB was not investigated in the manual phalanges (in white). The skeletal scheme used here is representative of the general bird skeleton depicting all considered elements, regardless of the species

**Fig. 4:**
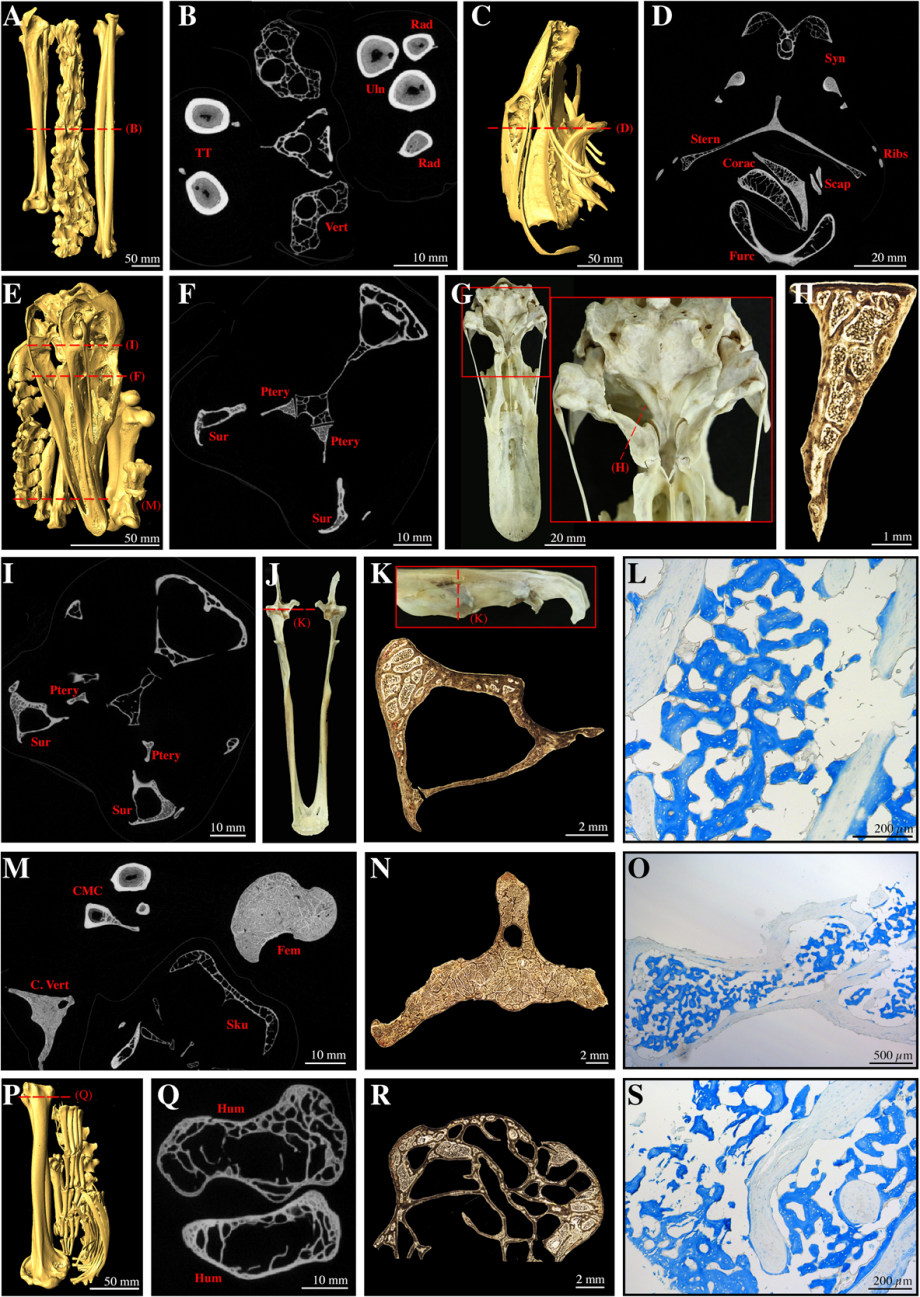
Medullary bone observed in the skeleton of *Cygnus atratus* (CM-S16508) using different techniques. **a**: isosurface of the tibiotarsi, vertebrae, radii, and ulnae; **b**: virtual cross-sections of different skeletal elements as shown in A. MB is filling the medullary cavity of the limb bones but is not seen in vertebral elements; **c**: isosurface of the synsacrum, keeled sternum, coracoids, scapulae, and furcula; **d**: virtual cross-sections of different skeletal elements as shown in C. MB is observed in all figured elements; **e**: isosurface of the skull, caudal vertebrae, carpometacarpii, femora, atlas, and axis; **f**: virtual cross-sections of the skull and mandible as shown in E. MB is visible in the cavities of the pterygoids and surangulars; **g**: photograph of the skull in ventral view, with higher magnification of the sampled pterygoid; **h**: Ground section of the pterygoid as shown in G; **i**: virtual cross-sections of the skull and mandible as shown in E. MB is visible in the cavities of the pterygoids and surangulars; **j**: photograph of the mandible; **k**: Ground section of the surangular as shown on higher magnification image. MB is visible in the cavities of the surangular; **l**: paraffin section of the surangular stained with Alcian Blue. MB is stained in dark blue, contrasting with the lightly stained surrounding trabecular bone, and indicates the distinct chemistries of the two bone types; **m**: virtual cross-sections of a cervical vertebra, the carpometacarpii, the skull, and a femur as shown in E. MB is visible in all elements; **n**: Ground section of a caudal vertebrae. The cavities are filled by MB; **o**: paraffin section of a caudal vertebra stained with Alcian Blue. MB is stained in blue and contrasts with the surrounding periosteal bone tissue that is lightly stained; **p**: isosurface of the humeri and other elements; **q**: virtual cross-sections of the humeral epiphyses as shown in P. MB is visible between the trabeculae. **r**: Ground section of a humeral epiphysis; **s**: paraffin section of the humeral ephiphysis stained with Alcian Blue. MB is stained in blue and contrasts with the surrounding trabecular bone tissue that is not stained. Abbreviations: CMC, carpometacarpus; Corac, coracoid; C. vert, caudal vertebra; Fem, femur; Furc, furcula; Hum, humerus; Ptery, pterygoid; Rad, radius; Scap, scapula; Sku, skull; Stern, sternum; Sur, surangular; Syn, synsacrum; TT, Tibiotarsus; Uln, ulna; Vert, vertebra

**Fig. 5:**
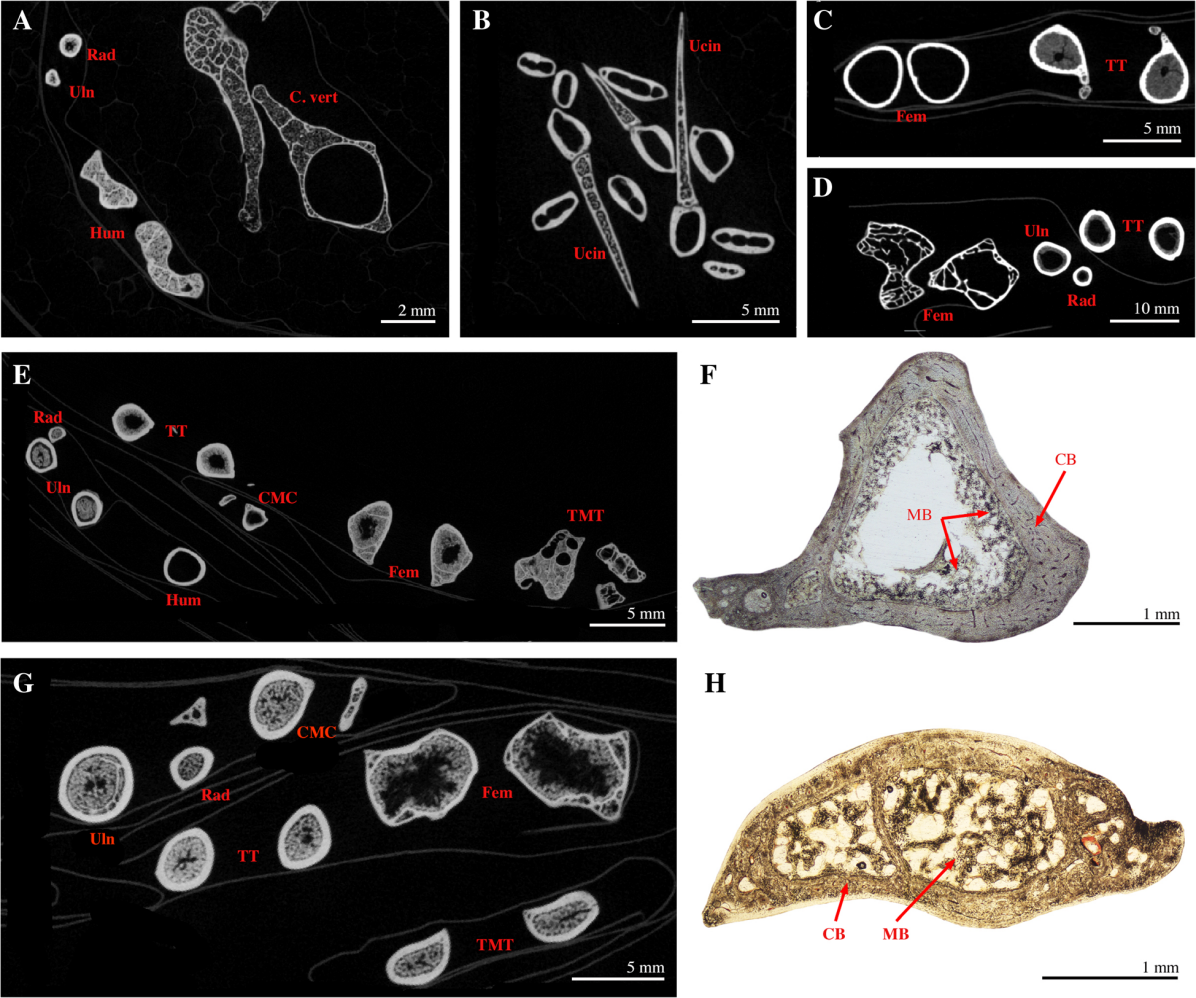
Virtual (**a-e, g**) and ground (**f, h**) cross-sections of skeletal elements in six bird species. **a**: MB is present in the minute skeletal elements (humerus, radius, ulna are shown here) of the hummingbird *Phaethornis superciliosus* (CM-S8888). Note that the un-pneumatized humeri contain large amounts of MB. The caudal vertebrae visible here belong to *Bonasa umbellus* (CM-S16804); **b**: MB is present in the uncinate processes, but not in the rib shafts of *Rhea americana* (YPM-148246); **c**: MB is present in the tibiotarsi, but not the femora of *Bonasa umbellus* (CM-S16804); **d**: MB is present in the tibiotarsi, but not the femora of *Micrastur ruficollis* (CM-S9081); **e**: MB is visible in different skeletal elements of *Cissa chinensis* (CM-S14901), including the tarsometatarsi; **f**: Mid-diaphyseal cross-section of the tarsometatarsus of *Cissa chinensis* (CM-S14901); **g**: MB is present in all featured limb bones of *Alisterus chloropterus* (CM-S16123), including the humeri (not shown) and tarsometatarsi; **h**: cross-section of the tarsometatarsus of *Alisterus chloropterus* (CM-S16123). Abbreviations: CB, cortical bone; CMC, carpometacarpus; C. vert, caudal vertebra; Fem, femur; Hum, humerus; MB, medullary bone; Rad, radius; TMT, tarsometatarsus; TT, tibiotarsus; Ucin, ucinate process; Uln, ulna

We document medullary bone in large quantities in all regions of the avian skeleton (including some elements of the cranium and mandible, see below and Figs. 3, 4 and 5), except the pedal phalanges (Fig. 3). Medullary bone can be deposited in the medullary cavity of long bone shafts and between trabeculae in the epiphyses (Figs. 4, 5), in the cavities of flat (e.g. girdle elements), short, and long bones (cf. [50]), and often fills large erosion rooms present in cortices. Finally, MB forms in large bones, as well as in minute skeletal elements such as the ribs of some small passerine birds (*Lonchura punctulata* TMM-M6977) or the bones of the hummingbird *Phaethornis superciliosus* (CM-S8888; Fig. 5a). Medullary bone is not consistent in its distribution across skeletal regions (Fig. 3), although it seems to be consistently absent or present in similar quantities contralaterally (e.g., right and left femora, or right and left pterygoids).

The prevalence of MB varies between the different regions of the axial skeleton. This tissue has been observed in the cranium and/or mandible in 57.1% of the specimens studied. Histochemical staining, which capitalizes on the chemical differences between MB and cortical/trabecular bone [15, 17] supports the visual identification of these tissues within the cavities of the pterygoid and the surangular of the black swan *Cygnus atratus* CM-S16508 (Fig. 4e-l), and in the pterygoid of the roseate tern *Sterna dougallii* CM-S7995 (not shown). MB is found in the atlas of 81.1% of the specimens, yet is far less prevalent in the axis of the sampled birds (only 54.3%). When found in the axis, MB is always present in the atlas, but the reverse is not true. Medullary bone is significantly less common in the rest of the cervical and thoracic vertebrae (28.9% of specimens). However, 78.9% of specimens exhibit MB within their caudal vertebrae/pygostyle. Medullary bone is common within costal elements (~ 58% in all or some ribs) even when these skeletal elements are minute (such as in some passerine birds). In *Rhea americana* (YPM-148246) and the great egret *Ardea alba* (CM-S13237), deposition of MB in the ribs is restricted to the ucinate processes (Fig. 5b).

Approximately half of our specimens possess MB in the sternum (47.4%) and in the rest of the pectoral girdle, including coracoids, scapulae, and the furcula. In the forelimb, MB is commonly present in the zeugopod (84.2% of all specimens), but not always in the same quantity between the radius and the ulna. Medullary bone is present in the carpometacarpus and the humerus of 37.8 and 31.6% of our specimens, respectively. When present in the carpometacarpus, MB is always found in the adjacent radius and ulna; but the reverse in not true. Only one specimen, the brown-headed cowbird *Molothrus ater* CM-S11856, presents small amounts of MB in the humerus and yet no MB (or just traces) in the rest of the forelimb elements.

Nearly half of the studied specimens possess MB in all or parts of the pelvic region including the synsacrum and the fused coxal elements (ilium, ischium, and pubis). MB can completely fill the cavities of caudal vertebrae/pygostyle and not be present in the synsacrum. However, when present in the synsacrum, it is always found in the region represented by the caudal vertebrae/pygostyle as well.

When formed, MB is universally found in the tibiotarsi (100% of the specimens; Fig. 3; Additional file 1: Table S1), and restricted to the proximal half of this element in most specimens. The second most common element to contain MB is the femur (94.7% of the specimens). However, MB was lacking in the femora of the falcon *Micrastur ruficollis* (CM-S9081; Fig. 5d) and the ruffed grouse *Bonasa umbellus* (CM-S16804; Fig. 5c); both of these presented nonetheless fair amounts of MB in 21.1 and 42.1% of the skeletal regions, respectively.

In the tarsometatarsus, MB is rare (21.6% of the sampled specimens), but when present, it can be deposited in great quantity, even in specimens of distantly related species that died at different stages of the laying cycle (e.g., the ticket tinamou *Crypturellus cinnamoneus* MCZ Orn 347981; the common green magpie *Cissa chinensis* CM-S14901, Fig. 5e, f; and the Papuan king parrot *Alisterus chloropterus* CM-S16123, Fig. 5g, h). Although rarely observed, the tarsometatarsus can contain MB even when the tissue is not prevalent throughout the rest of the skeleton. Indeed, only 38.9% of *Alisterus chloropterus* (CM-S16123) skeletal regions contain MB.

We also document a distal gradient in the deposition of MB throughout the length of the hind limb. When MB is observed in the tarsometatarsus, it is always present along the complete length of the tibiotarsus (from proximal to distal epiphyses). However, in most specimens (e.g., *Cygnus atratus*, CM-S16508 and *Laterallus leucopyrrhus*, CM-S11006) MB is restricted to the proximal part of the tibiotarsus and is absent entirely from the tarsometatarsus. This suggests that each hind limb element can produce a proximodistal depositional gradient, yet also that the entire hind limb functions as a single depositional unit, with the state of the distal limb elements contingent on the full deposition of MB throughout more proximal elements.

### Comparison between the skeletal distributions of MB and pneumaticity

For some species, we compared available data in the literature on the skeletal distribution of pneumaticity to that of MB (Additional file 2: Table S2). In several species, we found that some elements or skeletal regions reported as pneumatized by O’Connor ([48, 51]: Tables 2) contain either small or voluminous amounts of MB. We document that the atlas, keeled sternum, synsacrum, and coxal elements of *Anas luzonica* (TMM-M5395) and *Cygnus atratus* (CM-S16508) contain MB, although these elements are reported as pneumatized for both species [48, 51]. Likewise, some thoracic vertebrae, the coracoids, furcula, and humeral epiphyses of *Cygnus atratus* (CM-S16508) contain at least small amounts of MB (Fig. 4), despite the fact that air sac diverticula were directly observed in these elements in other black swan specimens [48]. Similarly, in five galliform species investigated by O’Connor [48], pneumatic postcranial elements generally included pre-caudal vertebrae (i.e., cervical, thoracic, and synsacral vertebrae), the sternum, and more variably other elements, but we document MB in the atlas and sternum of at least two galliform species (*Meleagris gallopavo* and *Colinus virginianus*). Previous studies have also shown that Strigiformes have extended postcranial pneumaticity [51], involving most of the vertebrae and girdle elements, yet Werning [43] observed MB in the coracoid of *Otus manadensis,* USNM-560674.

O’Connor ([51]: Fig. 4c) regarded members of the genus *Phaethon* as having a hyperpneumatized postcranial skeleton, and found pneumatic diverticula invading most skeletal elements, including distal forelimb segments, and the femur ([51]: table 2). Werning ([43]: appendix 2) reported the presence of MB in the ulna and femur of two different specimens of *Phaethon rubricauda* (USNM-622400, USNM-631988) using candling and direct visual observations*.* We CT-scanned the femur of *Phaethon rubricauda* (USNM-631988) and not only confirmed that it contains MB, but also that this specific bone tissue fills most of the shaft, as well as the inter-trabecular spaces in both epiphyses (Additional file 3: Figure S1).

Furthermore, we compared the presence/absence of osteological pneumatic features on the proximal ends of all sampled humeri and femora to the presence/absence of medullary bone in these elements (Additional file 4: Table S3). Femora of both specimens lacking MB (*Bonasa umbellus* CM-S16804 and *Micrastur ruficollis* CM-S9081) are pneumatized. By contrast, all femora containing MB in our sample are apneumatic (including *Phaethon rubricauda* USNM-631988). Similarly, all humeri lacking MB are pneumatized, whereas most humeri containing MB are apneumatic, except for two specimens (*Cygnus atratus* CM-S16508 and *Alisterus chloropterus* CM-S16123). These have pneumatized humeri with only small amounts of MB, yet the adjacent zeugopod is completely filled with MB in these specimens.

### Comparison between candling and CT-scan data

To compare the efficacy of candling [43], we micro-CT scanned the complete skeletons of nine specimens previously assessed using the candling method (Additional file 5: Table S4). We found some inconsistencies between results generated by the two approaches, specifically five false negatives in the results of Werning ([43]; see Additional file 5: Table S4). Werning [43] did not detect the presence of MB in the radius of *Limnodromus griseus* TMM-M8889 (Additional file 6: Figure S2A), in the scapula (not figured) and radius (Additional file 6: Figure S2B) of *Ptilinopus melanospilus* TMM-M11422, in the scapula of *Cardinalis cardinalis* TMM-M8909 (not figured), and in the tarsometatarsus of *Lonchura punctulata* TMM-M6977 (Additional file 6: Figure S2D), but CT examination showed the clear presence of this tissue.

## Discussion

### Ubiquitous distribution of MB

#### In the bird skeleton

We present the first taxonomically comprehensive assessment of MB skeletal distribution in Neornithes, and document that although the pattern and extent of MB skeletal distribution vary interspecifically, this ephemeral bone tissue is systemic in nature and can be deposited in all regions of the avian skeleton except the distal-most limb elements (pedal [and likely manual] phalanges; Fig. 3). The demonstrated commonality of MB in various skeletal regions conflicts with previous hypotheses that MB is predominantly formed in the medullary cavity of long limb bones [2, 42]. Although when present, MB is always found in the proximal half of the tibiotarsus, and in most cases, the femur (nearly 95% of the sampled specimens), and radius and ulna (84% of the sampled specimens), non-limb elements (e.g. pectoral and pelvic girdles, costal elements, select skull bones [pterygoid, surangular], and some vertebrae [atlas, axis, caudal vertebrae]) more frequently contain MB than do the remaining long bones (i.e., the humerus, tarsometatarsus, and carpometacarpus). MB is rarely observed in thoracic or most cervical vertebrae, with the exception of the atlas and axis, which contained MB in more than 40% of the studied specimens. Finally, MB was not observed in the (pedal) phalanges of any of the 40 sampled specimens, contrasting with Taylor and Moore [35], who recorded trace amounts in the pedal phalanges of laying pullets.

The prevalence of MB in a particular skeletal region is not related to the quantity of deposition in a given element. Although MB is infrequently deposited within the humerus, when present, it can form in great quantity, filling the medullary cavity as well as inter-trabecular spaces in the epiphyses. Finally, the hypothesis that the extent of MB skeletal distribution differs between wild and captive birds [43] is not supported by our results.

#### Across the bird phylogeny

Our data extend the known phylogenetic distribution of MB among Neornithes (Fig. 1). We report the first evidence of MB deposition in Piciformes, in a wild female downy woodpecker (*Dryobates pubescens*, CM-S8375) that died during lay. Candling data suggested the likely presence of MB in tyrant flycatchers (Tyrannidae) [43]; we confirm this using CT data from *Tyrannus verticalis* (TMM-M12926). MB is now documented in all of the major avian subclades (Palaeognathae, Galliformes, Anseriformes, Strisores, Columbaves, Gruiformes, Aequorlitornithes, Accipitriformes, Coraciimorphae, Australaves, cf. [52]; Fig. 1) suggesting it is ubiquitous in Neornithes. This, together with robust evidence for the presence of MB in fossil avialans [29, 32, 33] and a *T. rex* specimen [17, 20], support the hypothesis that this adaptation preceded the origin of Coelurosauria. However, we did not conduct an ancestral state reconstruction to determine the likelihood that the ancestral avian possessed MB, because the ephemeral nature of the tissue renders it difficult to confirm a species *inability* to deposit MB within our study design.

### Factors underlying the extent and pattern of MB skeletal distribution

Numerous studies have demonstrated that deposition and resorption of MB are induced by changes in steroid hormone levels (i.e., estrogen and androgen) during the egg-laying cycle, alternatively stimulating or inhibiting the activity of endosteal osteoblasts and osteoclasts [9, 41, 53–55]. Because the deposition and resorption of MB are controlled by specific hormone levels, we hypothesize a systemic ability of skeletal elements to form this bone tissue (i.e., hormones are distributed throughout the body by blood flow and thus all organs/skeletal elements that are estrogen receptive are expected to have a hormonally induced, tissue specific response). Our hypothesis is supported by observations on the extent of MB skeletal distribution across a broad taxonomic sample of wild bird species, as well as previous observations of estrogen-treated male pigeons, which showed MB in most skeletal elements [36]. Indeed, we show that MB commonly forms in all but the distal-most limb elements in the avian skeleton; however, we also document high interspecific variability in the extent and pattern of MB skeletal distribution that merits explanation. Two major, non-exclusive hypotheses have been put forth to account for the variation in the extent and pattern of MB distribution within the bird skeleton: blood supply and pneumaticity. Our data suggest that the distribution of MB can only be explained when both factors are taken together.

### Bone vascular supply and red marrow content

In their study, Landauer and Zondek [38] induced the deposition of MB in male ducks and chickens by exposing them to estrogen. In these species, skeletal elements responded differently to hormonal stimulus, with MB deposited in the hind limb segments along a proximodistal gradient. They observed MB within the femur and the tibiotarsus, but none was deposited in the tarsometatarsus, which is a poorly vascularized element in these birds. This deposition gradient was also observed within individual hind limb elements, where they showed that the proximal portion of the tibiotarsus contained high amounts of MB; however no MB was deposited distally. These authors also observed that MB deposition was strongly correlated to skeletal elements exhibiting degenerating red bone marrow. These observations led Landauer and Zondek [38] to propose that variation in MB deposition could be attributed to variation in blood supply, and thus differential exposure to hormones, and that MB primarily formed in well-vascularized bones containing hematopoietic marrow. Landauer and Zondek [38] also noted that the gradient observed in the hind limb was not present in the forelimb, presumably because the humerus was usually devoid of bone marrow in these species. They ascribed the lack of red bone marrow, and thus MB, to pneumaticity. Subsequent researchers reached the same conclusions by studying laying pullets, stating that in the absence of hematopoietic tissue and abundant blood supply, MB deposition cannot occur [35, 46, 47, 56].

**Fig. 6:**
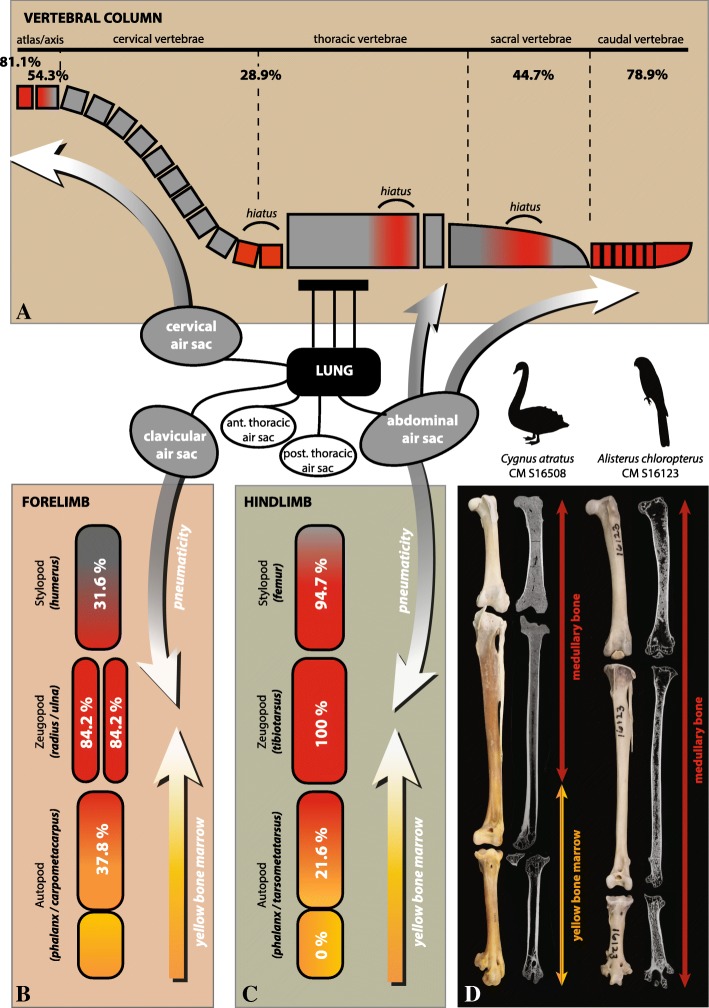
Skeletal distributions of pneumaticity (grey), yellow bone marrow (yellow), and MB/red bone marrow (red). **a**: along the vertebral column; **b**: along the forelimb; **c**: along the hind limb; **d**: The virtual longitudinal sections of the femur, tibiotarsus, and tarsometatarsus of two different individuals illustrate the proximo-distal gradient of MB deposition observed in the hind limb of most sampled specimens. When MB deposition is restricted to the proximal half of the tibiotarsus, it is always absent in the associated tarsometatarsus, as in *Cygnus atratus* (CM-S16508). When present in the tarsometatarsus, MB is always deposited along the complete shaft of the associated tibiotarsus, as in *Alisterus chloropterus* (CM-S16123)

Our data support the hypothesis that a proximodistal gradient of MB exists in the hind limbs (observed in 95% of sampled specimens) and forelimbs of extant birds (Fig. 6), and that this gradient is predicated on changes in vascularization and red marrow content within the limb. In our sample, MB is commonly deposited throughout the entire femur as well as the proximal half of the tibiotarsus, yet is rare in the tarsometatarsus, and absent in the pedal phalanges. Moreover, when present in the tarsometatarsus, MB is always distributed along the complete shaft of the associated tibiotarsus. Likewise, with one exception, when present in the humerus (31.6% frequency) MB is always present in the antebrachium (ulna/radius; 84.2% frequency), and all specimens containing MB in the carpometacarpus also contained MB in both adjacent zeugopod elements. These data provide strong support for a proximodistal gradient in the deposition of MB within the avian limb that is conserved across the avian phylogeny, yet exceptions to a consistent gradient occur. These revolve around the absence or only trace amounts of MB within the stylopod (femur and/or humerus) in some specimens.

For example, we observe the absence of MB within the femur of the banded forest falcon (falconiform) *Micrastur ruficollis* (CM-S9081) and the ruffled grouse (phasianid) *Bonasa umbellus* (CM-S16804). Unlike the majority of birds, the femora of some falconids and phasianids are pneumatized [43, 48, 51, 57–59]. We examined the femora of *Micrastur ruficollis* (CM-S9081) and *Bonasa umbellus* (CM-S16804) to determine if these specimens also exhibit femoral pneumaticity. We observed the presence of large osteological pneumatic features (see [60]: Fig. 1j) on the proximal epiphyses of these elements; thus, pneumaticity likely explains the absence of MB in the femur of these specimens (Additional file 4: Table S3). The femur of the only other sampled phasianid in our study, *Meleagris gallopavo* (NCSM-10222), contains MB and is apneumatic. A greater deviation from a consistent proximodistal gradient in MB deposition is observed within the forelimb (> 68% of humeri in our sample lack MB). Numerous studies have shown that the humerus is commonly pneumatized in birds [48, 51, 58, 59]. Our data supports this explanation. In our sample all pneumatic humeri lack MB, or exhibit only trace amounts. These results support the hypothesis that MB deposition is primarily controlled by vascularization (which generally decreases in more distal limb segments), with pneumaticity as a mitigating factor superimposed on this pattern (Fig. 6b-d).

### Skeletal pneumaticity

Birds inherited skeletal pneumaticity from their dinosaurian ancestors [61, 62]. Postcranial pneumaticity results from the progressive invasion of skeletal elements by diverticula of the pulmonary air sac system after hatching [48, 63–65]. As these diverticula invade the medullary cavity of skeletal elements, there is a simultaneous relocation and decrease in red marrow content [63, 65]. In pneumatized elements, the hematopoietic tissue eventually degenerates, yet small amounts can persist in the proximal and distal ends of the bones [66, 67]. Based on this relationship, researchers have speculated that the skeletal distributions of MB and pneumaticity should be inversely correlated, predicting only trace amounts or a complete lack of MB in pneumatized skeletal elements [44, 68]. We evaluated this hypothesis more widely across the avian skeleton (beyond our limb data, which we directly observed) by comparing the MB skeletal distribution data of the skull, axial column, and girdles with pneumaticity data available in the literature [48, 51].

For some of our specimens, we find that the skeletal distributions of MB and previously reported pneumaticity of skeletal regions other than the limbs overlapped, suggesting at first glance that our results did not support this proposed inverse relationship. We observed MB in the keeled sternum, synsacrum, and coxal elements of *Anas luzonica* (TMM-M5395) and *Cygnus atratus* (CM-S16508); elements previously reported as pneumatized for these species [48]. Similarly, the atlas and sometime the axis of several specimens of Galliformes (*Meleagris gallopavo*, *Colinus virginianus*, *Bonasa umbellus*) and Anseriformes (*Anas luzonica* and *Cygnus atratus*) contained MB, although O’Connor [48] identified the cranial cervical vertebrae of these species as pneumatized. A partial explanation for this discrepancy may arise from O’Connor [48, 51], who identified skeletal regions (e.g., cranial cervical vertebrae) as pneumatized when at least one element exhibited pneumaticity; thus not all cervical vertebrae must be invaded by diverticula to be declared pneumatized. Moreover, Hogg [69] investigated the distribution of pneumaticity along the vertebral column of more than a hundred adult specimens of domestic fowls, and observed that the cervical air sac rarely reached the axis and never invaded the atlas. Likewise, King ([70]: fig. 1) documented that of the 13 cervical vertebrae of six adult chickens, only the atlas and axis were apneumatic. This likely represents a common pattern among Neornithes and thus likely explains our observations that i) the atlas commonly contained MB in our sample, whereas the axis was less likely to contain MB and ii) MB was observed much less frequently in the remaining cervical vertebrae than in either of these elements (Figs. 3, 6a). Studies have also shown that the extent and pattern of skeletal pneumaticity is highly variable between closely related taxa, between adult individuals of the same species, and even contralaterally within a single skeletal element [69, 70]. King [70] observed that the extent of pneumatization of the keeled sternum and the pelvic girdle by the clavicular and abdominal air sacs respectively [48] varied between the six adult chickens examined. These data indicate that in some cases, the air sac diverticula incompletely invade the cavities of skeletal elements, thus, these could house some amounts of bone marrow. The presence of MB and pneumatization in a given skeletal element are not mutually exclusive.

**Fig. 7:**
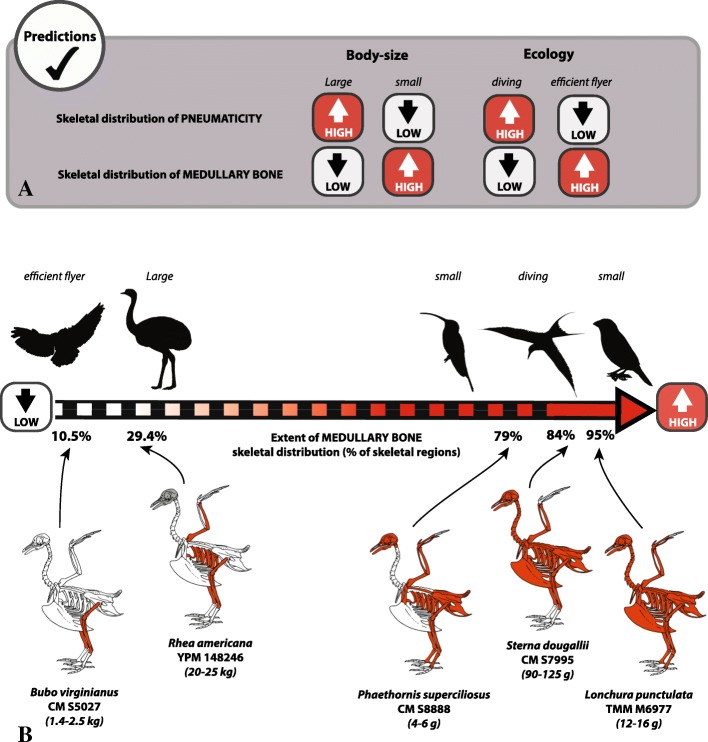
Relationship between the extents of MB and pneumaticity skeletal distributions. Predictions of MB skeletal distribution in birds with different body-sizes and ecologies if the inverse relationship between the skeletal distributions of MB and pneumaticity is verified (**a**). Diagram highlighting that our results support our predictions (**b**). Five selected specimens illustrate that MB skeletal distribution is relatively limited in large (e.g. *Rhea americana* YPM-148246) and active flying birds (e.g. *Bubo virginianus* CM-S5027), but extensive in small (*Phaethornis superciliosus* CM-S8888; *Lonchura punctulata* TMM-M6977) and plunge-diving birds (*Sterna dougallii* CM-S7995). Specific body mass data for adult females were gathered from [49]. The skeletal scheme used here is representative of the general bird skeleton depicting all considered elements, regardless of the species

Nonetheless, the vast majority of our MB distribution data is consistent with the skeletal pneumatization data found in the literature and with that we observed directly on our specimens (Additional file 4: Table S3). Several comparative studies have shown that the extent of avian skeletal pneumaticity varies inter-specifically, and is influenced by body-size and foraging ecology ([48, 51, 59, 60, 63, 71]; Fig. 7). Skeletal pneumaticity is typically limited in small bird species, regardless of flying ability (that usually exhibit “medullated” bones; [72]), yet extensive in large and efficient flyers (such as vultures, albatrosses, pelicans) and relatively well developed in large ratites [51, 63, 72, 73]. Moreover, several lineages of diving birds exhibit a reduction of postcranial pneumaticity [48, 51, 59, 60]. Thus, if skeletal pneumaticity is extensive in a given bird species, we would predict the inverse in MB distribution. Alternatively, we expect an expanded distribution of MB in very small, flying birds (Fig. 7). This relationship is somewhat supported by our dataset (Fig. 7). The hummingbird *Phaethornis superciliosus* (CM-S8888), the smallest bird in our sample, possesses an extensive distribution of MB, with ~ 79% of the skeletal regions containing MB, including large amounts of MB in the humerus and the tarsometatarsus. Werning ([43]: appendix 2) also observed an extensive distribution of MB in the hummingbird, *Microstilbon burmeisteri* (USNM-645254). The second smallest bird in our sample, *Lonchura punctulata* (TMM-M6977), exhibits the most extensive skeletal distribution of MB, with 94.7% of the bones containing at least small amounts of this tissue. *Rhea americana* (YPM-148246), the largest bird in our sample, possesses MB in only 29.4% of its postcranial skeleton. The most limited distribution of MB in our dataset (10.5% of the skeleton) is evidenced by *Bubo virginianus* (CM-5027), one of the largest efficient flyers examined. Its MB skeletal distribution (restricted to the femur and tibiotarsus) is congruent with the pneumatization pattern of Strigiformes described in O’Connor [51]. Both owl species studied by Werning [43] also showed a rather limited skeletal distribution of MB (Additional file 2: Table S2), congruent with our data. Finally, *Sterna dougallii* (CM-S7995), a plunge-diving bird, demonstrated wide skeletal distribution of MB (in 84.2% of the skeletal elements, including the non-pneumatized humerus).

Avian cranial pneumatization shows great inter-specific variation in extent and distribution pattern [69, 74]. However, Hogg [69] observed that in domestic fowl, pneumaticity is common in the neurocranium and the quadrate, yet more variable in the mandible and the pterygoid. Among our sample, when present in the skull, MB was always found in the pterygoid and/or surangular (part of the mandible).

Comparatives studies have shown that pneumaticity is common in avian cervical and thoracic vertebrae, yet less frequent in caudal vertebrae [48, 51, 61, 70, 73]. This distribution is inversely correlated to that of MB, which rarely occurs in cervical (except the atlas/axis complex) and thoracic vertebrae (only 28.9% of the sampled specimens), but is far more common in caudal vertebrae (78.9%; Fig. 6a). Similarly, the clavicular air sac often invades the humerus, yet rarely the more distal forelimb elements (i.e., radius, ulna, carpometacarpus; [48, 51, 57]). We find MB in the humerus of about a third of our sampled species, but only in small amounts in almost half of the cases. MB was more frequent in the distal forelimb elements. Finally, the femur and distal hind limb elements (tibiotarsus, tarsometatarsus, phalanges) are not commonly pneumatized [48, 51, 57]. This is congruent with the frequent presence of MB in the femur and the tibiotarsus. MB was nonetheless rare in the tarsometatarsus and absent in pedal phalanges, which are similarly non-pneumatized. It is important to note that MB should not necessarily be expected in non-pneumatized elements, because distal limb elements are also often poorly vascularized and do not contain red bone marrow but rather yellow fatty marrow [38, 65].

The first and only study comparing the development of pneumatization and the resulting distribution pattern of bone marrow after hatching within birds found that in adult pigeons, hematopoietic tissue was restricted to the ulna, radius, femur, tibiotarsus, scapula, furcula, and small portions of the keeled sternum and pelvic girdle ([65]: fig. 8E). In some apneumatic distal limb elements (carpometacarpus, tarsometatarsus, phalanges), red bone marrow had been converted to yellow fatty marrow. We sampled two species of Columbidae, closely related to the common pigeon. In both specimens, *Leptotila cassinii* (CM-S10149) and *Ptilinopus melanospilus* (TMM-M11422), the skeletal distribution of MB is positively correlated to the distribution of red bone marrow described by Schepelmann [65].

Our results confirm that MB forms in all non-pneumatized portions of the skeleton that contain red bone marrow (and are thus highly vascularized; Fig. 6). The extent and pattern of MB skeletal distribution thus depends on both skeletal pneumatization (indirectly related to specific body size and foraging ecology), and the presence of red bone marrow.

### Reproductive biology

Our data do not support a direct, exclusive relationship between the skeletal distribution of MB and clutch size. Species laying small clutches can exhibit extensive MB skeletal distribution. *Phaethornis superciliosus* (CM-S8888) and *Sterna dougallii* (CM-S7995) have an average clutch size of two eggs, and yet exhibit MB in 78.9 and 84.2% of the skeletal elements, respectively. On the other hand, species with larger clutch sizes can exhibit a more limited skeletal distribution of MB. For example, the tinamou *Crypturellus boucardi* can lay up to 10 eggs in the same clutch [49]. Specimen MCZ-Orn336971 died at the beginning of the laying cycle (with at least two yolky follicles), yet possessed MB in only 52.6% of skeletal regions considered.

However, experimental data suggest a possible relationship between clutch size and *quantity* of MB deposited. Landauer et al. [37] exposed two different breeds of duck to the same dose of estradiol benzoate and recorded different quantities of MB deposition that appear to correlate with clutch size (high MB deposition in the Pekin duck, which lays > 150 eggs per year [75] and low MB deposition in mallards that lay 9–13 eggs per clutch [49]). Although it is reasonable to propose a relationship between total MB volume and amount of mineral mobilization required for eggshell formation, if a correlation exists, it is likely to be compounded by a variety of other biotic factors including eggshell mineral density, clutch size, eggshell volume, rapidity of sequential egg-laying, body size, cortical thickness, and/or extent of skeletal pneumaticity (i.e., amount of open spaces left to deposit MB).

### Implications for the identification of MB in extinct archosaurs

Medullary bone-like tissues have been reported in several extinct avemetatarsalian specimens based on a set of microstructural, chemical, and developmental criteria recognized to be specific to MB [2, 17, 18, 20]. However, the current diagnosis of MB is described primarily from that found in gravid females or hormonally treated males of domestic birds; thus is not representative of Neornithes diversity. Several recent studies demonstrate that some avian bone pathologies meet some of the criteria commonly used to define MB [19, 34, 76], and provide an alternative for previous observations of MB-like tissues in the fossil record. The extent and pattern of MB skeletal distribution in wild bird species has not previously been documented, and several neontological and paleobiological studies [20, 34] or even reviews [2] have presumed that MB is mostly formed in long limb bones, without actually testing this assumption. As a consequence, the nature of MB-like tissues found in some extinct avemetatarsalians has been questioned based, in part, on their apparent unusual anatomical location [16, 19, 23, 34]. Other criteria are clearly needed to diagnose these tissues; criteria likely to survive in the fossil record.

Here we document that MB can form systemically within the avian postcranial skeleton and that its distribution is dictated by the distribution of pneumatic diverticula and red bone marrow. Moreover, a few elements of the skull do form MB during the reproductive cycle of some female birds, as previously noted for laying pullets [35]. Therefore, “unusual” anatomical location of MB-like tissues found in extinct avemetatarsalians is not sufficient to exclude a diagnose of this tissue and its potential reproductive nature.

Skeletal pneumaticity has been reported to various degrees in several groups of non-avian dinosaurs [61, 77–82] and pterosaurs [83]. Pneumaticity is mainly confined to the vertebral column and ribs in sauropodomorph and theropod dinosaurs [61, 78, 79], yet does invade the pelvic girdle in select species of both clades [77, 84, 85], and the forelimb elements in theropod dinosaurs, only rarely invading the hindlimbs [77]. As in modern birds, it is absent or limited to the vertebral column in small (and more basal) pterosaurs, yet can be extensive in larger, later diverging taxa. Thus, some Pterodactyloidea subclades also exhibit pneumatic girdles and limb elements [86].

Our results confirm that skeletal distribution of MB is directly related to the distribution of red bone marrow, and inversely correlated to the combined skeletal distributions of pneumaticity and yellow bone marrow. Therefore, the pneumatization of skeletal elements in extinct avemetatarsalians, usually assessed by the presence of unambiguous osteological, structural (i.e., pneumatic foramina leading to large internal cavities; [77]), and histological correlates (i.e., pneumosteal tissue sensu Lambertz et al. [87]), could be considered as an additional source of information (besides the usual microstructural, chemical, and developmental criteria established in previous studies; [16–19, 33]) to reassess the nature of MB-like tissues. However, this criterion would have to be considered cautiously and never on its own. Indeed, studies have shown that in some cases, air sac diverticula do no completely invade the cavities of pneumatized skeletal elements and small amounts of bone marrow, and hence MB, can persist (e.g., present study; [66, 67, 70]). Several studies have shown that the complexity and organization of the air sac system known from modern birds already existed in saurischian dinosaurs [61, 79, 82]. Thus, at least in sauropods and non-avian theropods, data suggest that the cervical vertebrae are invaded and pneumatized (from caudal ones to the more cranial ones) by cervical air sac diverticula. Likewise, abdominal air sac diverticula pneumatize the sacral (and potentially caudal) vertebrae [61, 79, 82]. As in birds, the humerus and pectoral girdle elements of some saurischians can potentially be pneumatized by diverticula of the clavicular airsac. Finally, the femur is sometime pneumatized by abdominal air sac diverticula ([48, 61, 82, 85]; Fig. 6).

The correlation we demonstrate between MB distribution and pneumaticity, and the presence of an air sac system homologous to modern birds in saurischian dinosaurs, allow us to predict aspects of MB skeletal distribution in sauropods and non-avian theropods: i) if purported MB-like tissues are found within any skeletal element of a non-avian dinosaur specimen, MB should systematically be identified within at least the proximal tibia of this individual (and in the contralateral bone in case of paired elements); ii) if found in the skull, MB-like tissues should be present in at least the pterygoid and/or the surangular; iii) if found in the autopod, MB-like tissues should also be present in the adjacent zeugopod; iv) if found in the axis, MB-like tissue should also be present in the atlas; v) if found in any cervical vertebrae (except within the cervicodorsals), MB-like tissues should also be present in the atlas and axis; and vi) when found in the most caudal sacral vertebrae, MB-like tissues should also be present in caudal vertebrae. These criteria can be used to refine arguments on the validity of MB-like tissues in avemetatarsalians.

## Conclusions

The present work constitutes the first comprehensive investigation of MB skeletal distribution across the bird phylogeny, using μCT and histochemical data, and revisits previous hypotheses pertaining to MB distribution patterns.

We document that the skeletal distribution of MB varies interspecifically, but does not differ between captive and wild-caught individuals. We find MB is a systemic tissue that can be deposited within virtually all skeletal regions, including cranial elements, and note that it is uniformly present in the proximal tibiotarsus of all studied specimens (Fig. 3). Thus, we find anatomical location of purported MB in extinct archosaurs to be an invalid criticism against the potential reproductive nature of these tissues.

Our results confirm previous hypotheses that skeletal distribution of MB is directly related to the distribution of red bone marrow, and inversely correlated to the combined skeletal distributions of pneumaticity and yellow bone marrow (Fig. 6). The extent of pneumaticity skeletal distribution is linked to the body size and lifestyle habits of bird species. Hence we find that small-bodied and some diving birds possess widespread deposition of MB, whereas MB distribution is highly restricted in large-bodied or efficient flyers (Fig. 7).

The proposed homology of the pulmonary system between living birds and some non-avian dinosaurs permit us to derive a series of location-based predictions that can be used to critically evaluate purported MB-like tissues in fossil specimens.

Finally, we find candling to be a rapid, effective method for screening for MB in skeletal collections; yet note a moderate amount of type II errors with this approach.

## Material & Methods

### Biological sample

Our skeletal sample encompasses a broad array of avian diversity, including 40 specimens representing 38 species (Fig. 1; Additional file 1: Table S1). Sampled individuals span over three orders of magnitude in adult body mass (4–25,000 g), represent a range of ecologies (e.g., diurnal/nocturnal, terrestrial/semiaquatic, migratory), reproductive strategies (e.g., avg. clutch size between 1 and 40), and reproductive status (stage within the lay-cycle). These include a large ratite (the greater rhea *Rhea americana*, YPM-148246), medium-sized birds (e.g., black swan *Cygnus atratus*, CM-S16508; wild turkey *Meleagris gallopavo*, NCSM-10222), and diminutive species (e.g., the hummingbird *Phaethornis superficialis,* CM-S8888), as well as i) fully terrestrial birds (e.g., the greater rhea); ii) mostly terrestrial birds (e.g., the tinamous and select galliformes); iii) water birds (e.g., select members of Anatidae and Aequorlitornithes); iv) and efficient flyers. The range of ecological parameters for each species is tabulated in Additional file 1: Table S1. Our phylogenetic scope is equally broad, comprised of seven palaeognaths, and the majority of neognath subclades (as defined by [52]), including three Galliformes, two Anseriformes, two Strisores, three Columbaves, a single Gruiformes, six Aequorlithornithes, two Strigiformes, two Coraciimorphae, and 10 Australaves (including seven Passeriformes; see Fig. 1, Additional file 1: Table S1). More than 70% of our sample is constituted by wild-caught individuals.

To amass a skeletal sample of female birds that perished during the laying cycle, we screened through hundreds of specimens hosted in the collections of six different institutions in the United States of America: Carnegie Museum of Natural History, Pittsburgh, PA (CM); the Museum of Comparative Zoology, Department of Ornithology, Harvard University, Cambridge, MA (MCZ); Museum of Vertebrate Zoology, University of California, Berkeley, CA (MVZ); North Carolina Museum of Natural Sciences, Ornithology Unit, Raleigh, NC (NCSM); Texas Vertebrate Paleontology Collections, University of Texas, Jackson School Museum of Earth History, Austin, TX (TMM); Peabody Museum of Natural History, Yale University, New Haven, CT (YPM). Specimens were selected based on data (when recorded) on the state of the reproductive tract at the time of death (see Additional file 1: Table S1) and/or by candling the major limb bones following the method developed by Werning [43]. Bones with an empty medullary cavity are expected to candle translucent, whereas bones filled with MB should appear opaque. Nevertheless, the use of this method may result in false positives, when the studied skeletal elements had not been previously drained of their marrow and other soft tissue contents or had thick cortices (see [43] for details).

Medullary bone is an ephemeral tissue whose formation and subsequent resorption in the cavities of skeletal elements follow the ovarian estrogen cycle [2, 38, 88]. Thus, MB is formed in female birds shortly before the onset of the laying cycle, during the maturation of the ovarian follicles and is partly or completely resorbed towards the end of the cycle during the eggshell calcification. Therefore, knowing that the female died during the laying cycle does not necessarily mean that it still presents MB in its skeletal elements. Indeed, if the individual was at the end of the laying period, MB may have already been completely resorbed to form the last egg of the clutch [2]. Moreover, because the amount of medullary bone within the skeleton is known to vary throughout the lay cycle we would expect it to vary within a single individual depending on the timing of sampling.

Heterogeneous collection database records across the various institutions render it difficult to assign all specimens to a prescribed stage in the laying cycle. However, available collection data was used to gather specimens that clearly represent various stages of egg formation. Some specimens died at the beginning of the cycle during the maturation of the ovarian follicles (e.g., *Rhea americana* YPM-148246). Several died with an incompletely formed egg in the oviduct; whereas others were collected during a stage close to oviposition, but not necessarily at the end of the laying period, with a fully formed shelled-egg in the oviduct (e.g., *Crypturellus cinnamomeus* MCZ-Orn 347981 and *Aethia pusilla* CM-S15857). Information about the state of the reproductive tract was available for 33 of the considered specimens (Additional file 1: Table S1). Some variation in the skeletal distribution of MB in our sample might thus be attributable to differential sampling within the window of deposition and subsequent resorption.

### Methods

To investigate the skeletal distribution of MB, we scanned the (sub-) complete skeleton of each selected specimen with a high-resolution μCT scanner (Nikon XTH 225 ST) at the Shared Materials Instrumentation Facility of the Duke University, Durham NC, USA. The resolution of our μCT scans ranged from 17.5 to 106.8 μm, depending on the size of the specimens (Additional file 7: Table S5). All μCT scans supporting the results of this article are available in the MorphoSource repository, under the project # P640 at https://www.morphosource.org/Detail/ProjectDetail/Show/project_id/640 [89].

Data were imported into Avizo Lite (version 9.0.0) for visualization. For each specimen, we recorded the presence/absence of MB in 19 predetermined bones or skeletal regions encompassing the complete skeleton (see Additional file 1: Table S1; Figs. 2, 3) and defined as follows: 1) skull (all elements were scored as a single skeletal unit including the cranium and mandibles), 2) atlas, 3) axis, 4) all other cervical and thoracic vertebrae, 5) sternum, 6) coracoid, 7) scapula, 8) furcula, 9) costal elements (excluding fused cervical ribs, these were scored with respective vertebrae), 10) humerus, 11) radius, 12) ulna, 13) carpometacarpus, 14) synsacrum and fused coxal elements (illium, ischium, pubis), 15) caudal vertebrae/pygostyle, 16) femur, 17) tibiotarsus, 18) tarsometatarsus, 19) pedal phalanges.

Because the amount of MB present in a given skeletal element, as well as its rate of mineralization can vary during the lay cycle [10, 41], the aspect and microstructure of MB can likewise vary between specimens on virtual sections (Additional file 6: Figure S2). However, mineralized MB was easily distinguishable from other tissue types, because it always appears as dense or less dense than the surrounding cortical and trabecular bone (Figs. 4, 5, Additional file 6: Figure S2). Moreover, MB has a “granular” and heterogeneous texture due to its trabecular meshwork architecture.

In our sample, a few specimens included pathological skeletal elements (e.g., one ulna of *Colinus virginianus* TMM-M6536), but these bones were easily identified by their abnormal external morphology. The associated endosteal pathological bone tissues were also clearly distinct from MB based on a different mineral:organic ratio and overall microstructure (see Additional file 6: Figure S2C). Finally, soft tissues remaining on incompletely prepared skeletons (keratin, tendons, skin, etc.) and in the cavities of non-drained skeletal elements (e.g. marrow) are distinct from bone tissues, and show a smooth and homogeneous aspect (Additional file 6: Figure S2B).

Furthermore, MB is always deposited and mineralized centripetally, from the endosteal margin to the center of the cavity [14, 39, 88]. When present in small amounts, MB universally contacts the endosteal margin of cavities and trabeculae (Additional file 6: Figure S2C, D). When only a thin layer of MB was observed, this information was reported in Additional file 1: Table S1.

We conducted additional analyses to ascertain the nature of purported MB identified via μCT scanning, using histochemical techniques. These included instances of purported MB in controversial locations (e.g. in some cranial elements, or in the tarsometatarsus) or diminutive elements (e.g. the minute ribs of some bird species). For direct visual observation of endosteal tissues, we processed ground-sections following standard petrographic protocols [90] in the Paleontology Research Lab of the North Carolina Museum of Natural Sciences, Raleigh, NC. The bone fragments were dehydrated in progressive alcohol baths (70 to 100%), defatted in acetone, and embedded in a clear polyester casting resin (EPO-TEK 301). A slice of embedded bone was cut with a Buehler IsoMet 1000 Precision Saw, affixed to a glass slide with epoxy, and ground to desired thickness (100–80 μm) with a Buehler MetaServ 250 Grinder**-**Polisher. Ground-sections were observed with a Nikon Eclipse C*i* POL microscope equipped with a polarizer and a lambda filter, and imaged with a Nikon DS-Fi2 digital camera.

Select specimens underwent additional testing via Alcian Blue or High Iron Diamine (HID), following the protocols detailed in Schweitzer et al. [17] to confirm the presence of MB. Alcian Blue or HID can differentiate the type and amount of glycosaminoglycans incorporated into various bone matrices and have been used to diagnose MB and discriminate between MB and surrounding cortical and trabecular bone [15]. Bone samples were fixed with neutral buffered 10% formalin overnight, then demineralized in 500 mM EDTA (pH 8.0) until all mineral was removed, and again subjected to fixation as above. Demineralized samples were then subjected to dehydration (via sequential incubations in 70, 80, 90, 95, and 100% alcohols for ~ 1 h each), followed by three, 30-min incubations in 100% xylene to clear the tissue. Tissues were then transferred to three separate and sequential incubations in 100% paraffin for 30 min each, to complete infiltration, and finally embedded in paraffin wax (Paraplast Plus EMS) for sectioning. Sections were taken at 5 μm, using a Leica RM 2255 microtome. Paraffin embedded sections were deparaffinized with xylene, and dehydrated through a graded ethanol series. The demineralized bone sections were either stained in Alcian Blue for 30 min, or oxidized in 1% periodic acid for 10 min, rinsed under running tap water, then incubated with freshly made HID solution overnight. Sections were then rinsed with tap water, dehydrated with a graded ethanol series, followed by three incubations in 100% xylene, and mounted with a mounting medium (Poly**-**Mount, PolySciences) and cover glass for visualization. Paraffin sections were examined with a Zeiss Axioskop 2 microscope and imaged using an AxioCam MRc 5 (Zeiss) digital camera and the Axiovision software package (version 4.7.0.0). All paraffin sections and chemical staining analyses were conducted in the Molecular Paleontology laboratory of the Biological Sciences Department, North Carolina State University, Raleigh, NC. A list of specimens treated with these protocols is provided in Additional file 8: Table S6.

To reassess previous hypotheses pertaining to the skeletal distribution of MB, we collected information from the literature about the skeletal pneumatization of some bird species ([48, 51, 58]; Additional file 2: Table S2). We also recorded the presence/absence of osteological pneumatic features on the proximal ends of all sampled humeri and femora, to compare the presence of pneumaticity and medullary bone in these elements (Additional file 4: Table S3). Humeri were coded as pneumatized if a large pneumatic foramen was observed on the caudal aspect (e.g., see [48]: Fig. 4). Femora were coded as pneumatized if a large, circular pneumatopore was observed on the craniomedial side of trochanter femoris (see [60]). Finally, for a few specimens we compared our CT-data with the results obtained by Werning [43] using the candling method on the exact same individuals (see Additional file 5: Table S4).

## Additional files

Additional file 1: **Table S1.** Dataset comprising information on 1) the 40 specimens sampled; 2) their skeletal distribution of MB. The presence (√) or absence (X) of MB was recorded in 19 skeletal elements / regions representing most of the bird skeleton (manual phalanges nor included in the present study); 3) the history traits and ecology of the sampled species. See Material and Methods section for institutional abbreviations. (XLS 88 kb)

Additional file 2: **Table S2.** Comparison of the skeletal distributions of MB and pneumaticity in different bird species. Data on the skeletal distribution of MB come from the present study and the study of Werning ([43]: appendix 2). Data on the skeletal distribution of pneumaticity have been compiled from O’Connor ([48]: table 2), and O’Connor ([51]: table 2). The presence (√) or absence (X) of MB and pneumaticity was recorded in different elements / regions of the bird skeleton. We highlighted in red skeletal elements considered as pneumatic and containing MB. (XLS 41 kb)

Additional file 3: **Figure S1.** Virtual longitudinal section of the femur of *Phaethon rubricauda* (USNM-631988). MB fills up most of the medullary cavity, as well as inter-trabecular spaces in both epiphyses. (JPG 486 kb)

Additional file 4: **Table S3.** Comparison of the distributions of MB and pneumaticity in the stylopod of all studied specimens. The presence (√) or absence (X) of osteological pneumatic features on the proximal epiphyses of the humerus and femur was recorded for each sampled specimen and confronted to the dataset on the presence (√) or absence (X) of MB in these elements. Conflicting results are highlighted in red. (XLS 52 kb)

Additional file 5: **Table S4.** Comparison of candling [43] and μCT data (the present study) for selected specimens. All specimens are from the Texas Vertebrate Paleontology Collections, University of Texas, Jackson School Museum of Earth History, Austin, TX (TMM). The presence (√) or absence (X) of MB was recorded in different elements / regions of the bird skeleton. Conflicting results are highlighted in red. (XLS 37 kb)

Additional file 6: **Figure S2.** Virtual cross-sections of limb bones in four different bird species. **A:** *Limnodromus griseus* (TMM-M8889). MB is present in the medullary cavities of all figured skeletal elements, including the humeri; **B:** *Ptilinopus melanospilus* (TMM-M11422). In this specimen, MB is not present in the humeri, carpometacarpi, and tarsometatarsi. As in all other specimens, MB has a granular aspect, while soft tissues appear smooth. Both femora show an unusual and very dense endosteal bone tissue (probably pathological) that fills up part of the medullary cavity, but is easily distinguishable from the neighboring MB; **C:** *Colinus virginianus* (TMM-M6536). In this specimen, MB is not present in the humeri, carpometacarpi, and tarsometatarsi. One ulna presents a pathology, with a periosteal pathological bone tissue and an endosteal pathological bone tissue that is clearly distinguishable from MB, based on mineralization rate and microstructure; **D:** *Lonchura punctulata* (TMM-M6977). MB is present in the medullary cavities of all figured skeletal elements, including the humeri and tarsometatarsi, although in very small quantities. **E:** *Anas luzonica* (TMM-M5395). MB is not present in the humeri of this specimen, but is clearly visible in the carpometacarpi. Abbreviations: CMC, carpometacarpus; Fem, femur; Hum, humerus; MB, medullary bone; PB, pathological bone; Rad, radius; ST, soft tissue; TT, tibiotarsus; TMT, tarsometatarsus; Uln, ulna. (JPG 2509 kb)

Additional file 7: **Table S5**. List of μCT scans used in the present study. All μCT scans are available in the MorphoSource repository, under the project # P640 at https://www.morphosource.org/Detail/ProjectDetail/Show/project_id/640 [89]. (XLS 60 kb)

Additional file 8: **Table S6**. Subsample of specimens used for destructive analyses (petrographic and paraffin thin-sections, and chemical staining). (XLS 32 kb)

## Abbreviations

CM: Carnegie Museum of Natural History, Pittsburgh, PA
MB: Medullary bone
MCZ: The Museum of Comparative Zoology, Department of Ornithology, Harvard University, Cambridge, MA
MVZ: Museum of Vertebrate Zoology, University of California, Berkeley, CA
NCSM: North Carolina Museum of Natural Sciences, Ornithology Unit, Raleigh, NC
TMM: Texas Vertebrate Paleontology Collections, University of Texas, Jackson School Museum of Earth History, Austin, TX
USNM: National Museum of Natural History, Smithsonian Institution, Washington, DC
YPM: Peabody Museum of Natural History, Yale University, New Haven, CT
μCT: Micro computed-tomography

## References

[1] Schweitzer MH, Elsey RM, Dacke CG, Horner JR, Lamm ET (2007). Do egg-laying crocodilian (*Alligator mississippiensis*) archosaurs form medullary bone?. Bone..

[2] Dacke CG, Arkle S, Cook DJ, Wormstone IM, Jones S, Zaidi M, Bascal ZA (1993). Medullary bone and avian calcium regulation. J Exp Biol.

[3] Buckner GD, Martin JH, Hull FE (1930). The distribution of blood calcium in the circulation of laying hens. Am J Physiol-Legacy Content.

[4] Mueller WJ, Schraer R, Scharer H (1964). Calcium metabolism and skeletal dynamics of laying pullets. J Nutr.

[5] MacLean SF (1974). Lemming bones as a source of calcium for arctic sandpipers (*Calidris* spp.). Ibis.

[6] Graveland J, Gijzen TV (1994). Arthropods and seeds are not sufficient as calcium sources for shell formation and skeletal growth in passerines. Ardea.

[7] Foote JS (1911). The comparative histology of femoral bones. Trans Am Microsc Soc.

[8] Foote JS. A contribution to the comparative histology of the femur. Smithsonian Contributions to Knowledge. 1916;35:1–230.

[9] Kyes P, Potter TS (1934). Physiological marrow ossification in female pigeons. Anat Rec.

[10] Kerschnitzki M, Zander T, Zaslansky P, Fratzl P, Shahar R, Wagermaier W (2014). Rapid alterations of avian medullary bone material during the daily egg-laying cycle. Bone..

[11] Bonucci E, Gherardi G (1975). Histochemical and electron microscope investigations on medullary bone. Cell Tissue Res.

[12] Candlish JK, Holt FJ (1971). The proteoglycans of fowl cortical and medullary bone. Comp Biochem Physiol B Comp Biochem.

[13] Fisher LW, Schraer H (1980). The glycosaminoglycans of estrogen-induced medullary bone in Japanese quail. Arch Biochem Biophys.

[14] Schraer H, Hunter SJ (1985). The development of medullary bone: a model for osteogenesis. Comp Biochem Physiol A Physiol.

[15] Yamamoto T, Nagaoka N, Hirata A, Nakamura H, Inoue M, Kawai M, Ikegame M (2005). Ultrastructural and immunohistochemical studies of medullary bone calcification, with special reference to sulphated glycosaminoglycans. J Electron Microsc.

[16] Chinsamy A, Cerda I, Powell J (2016). Vascularised endosteal bone tissue in armoured sauropod dinosaurs. Sci Rep.

[17] Schweitzer MH, Zheng W, Zanno L, Werning S, Sugiyama T (2016). Chemistry supports the identification of gender-specific reproductive tissue in *Tyrannosaurus rex*. Sci Rep.

[18] Werning S, Schweitzer M, Padian K (2016). Microstructure isn’t enough: additional diagnostic criteria to test among hypotheses of bone tissue identity. Anat Rec.

[19] Prondvai E (2017). Medullary bone in fossils: function, evolution and significance in growth curve reconstructions of extinct vertebrates. J Evol Biol.

[20] Schweitzer MH, Wittmeyer JL, Horner JR (2005). Gender-specific reproductive tissue in ratites and *Tyrannosaurus rex*. Science..

[21] Sato T, Cheng YN, Wu XC, Zelenitsky DK, Hsiao YF. A pair of shelled eggs inside a female dinosaur. Science. 2005;308:375–375. 10.1126/science.1110578 15831749

[22] Chinsamy A, Codorniú L, Chiappe L (2009). Palaeobiological implications of the bone histology of *Pterodaustro guinazui*. Anat Rec.

[23] Prondvai E, Stein KH (2014). Medullary bone-like tissue in the mandibular symphyses of a pterosaur suggests non-reproductive significance. Sci Rep.

[24] Lee AH, Werning S (2008). Sexual maturity in growing dinosaurs does not fit reptilian growth models. PNAS..

[25] Hübner TR (2012). Bone histology in *Dysalotosaurus lettowvorbecki* (Ornithischia: Iguanodontia)–variation, growth, and implications. PLoS One.

[26] Cerda IA, Pol D (2013). Evidence for gender-specific reproductive tissue in a basal sauropodomorph dinosaur from the late Triassic of Argentina. Ameghiniana..

[27] Tremaine K, Woodward Ballard H, Horner JR. Bone histology of an immature *Tyrannosaurus rex* with comments on unusual endosteal bone tissue. J Vert Paleontol, Program and Abstracts. 2014;240:240.

[28] Skutschas PP, Boitsova EA, Averianov AO, Sues HD (2017). Ontogenetic changes in long-bone histology of an ornithomimid theropod dinosaur from the upper cretaceous Bissekty formation of Uzbekistan. Hist Biol.

[29] Chinsamy A, Chiappe LM, Marugán-Lobón J, Chunling G, Fengjiao Z (2013). Gender identification of the Mesozoic bird *Confuciusornis sanctus*. Nat Commun.

[30] Smith NA, Clarke JA (2014). Osteological histology of the pan-Alcidae (Aves, Charadriiformes): correlates of wing-propelled diving and flightlessness. Anat Rec.

[31] Angst D, Chinsamy A, Steel L, Hume JP (2017). Bone histology sheds new light on the ecology of the dodo (Raphus cucullatus, Aves, Columbiformes). Sci Rep.

[32] O’Connor JK. Definitive occurrence of medullary bone in an enantiornithine (Aves: Ornithothoraces). ISPH 2017 Abstract book. p.78.

[33] O’Connor J, Erickson GM, Norell M, Bailleul AM, Hu H, Zhou Z. Medullary bone in an early cretaceous enantiornithine bird and discussion regarding its identification in fossils. Nat Commun. 2018. 10.1038/s41467-018-07621-z. 10.1038/s41467-018-07621-z PMC6281594 30518763

[34] Cerda IA, Chinsamy A, Pol D (2014). Unusual endosteally formed bone tissue in a Patagonian basal sauropodomorph dinosaur. Anat Rec.

[35] Taylor TG, Moore JH (1953). Avian medullary bone. Nature..

[36] Clavert J, Benoit J (1942). Enrichissement du squelette en calcium chez le Pigeon sous l'action du dipropionate d'œstradiol. CR Soc Biol Paris.

[37] Landauer W, Pfeiffer CA, Gardner WU, Shaw JC (1941). Blood serum and skeletal changes in two breeds of ducks receiving estrogens. Endocrinology.

[38] Landauer W, Zondek B (1944). Observations on the structure of bone in estrogen-treated cocks and drakes. Am J Pathol.

[39] Bloom MA, Domm LV, Nalbandov AV, Bloom W (1958). Medullary bone of laying chickens. Am J Anat.

[40] Ankney CD, MacInnes CD (1978). Nutrient reserves and reproductive performance of female lesser snow geese. Auk.

[41] Squire ME, Veglia MK, Drucker KA, Brazeal KR, Hahn TP, Watts HE (2017). Estrogen levels influence medullary bone quantity and density in female house finches and pine siskins. Gen Comp Endocrinol.

[42] Whitehead CC (2004). Overview of bone biology in the egg-laying hen. Poult Sci.

[43] Werning S (2018). Medullary bone is phylogenetically widespread and its skeletal distribution varies by taxon. J Ornithol.

[44] Rick AM (1975). Bird medullary bone: a seasonal dating technique for faunal analysis. Bull Can Archaeol Assoc.

[45] Benoit J, Clavert J (1945). Action ostéogénique directe et locale de la folliculine, démontrée, chez le canard et le pigeon, par son introduction localisée dans un os long. C R Seances Soc Biol Fil.

[46] Taylor TG, Moore JH, Loosmore RM (1958). Some effects of bone fracture in hens. Zentralbl Veterinarmed.

[47] Taylor TG, Moore JH (1956). The effect of calcium depletion on the chemical composition of bone minerals in laying hens. Brit J Nutrition.

[48] O'Connor PM (2004). Pulmonary pneumaticity in the postcranial skeleton of extant Aves: a case study examining Anseriformes. J Morphol.

[49] Handbook of the Birds of the World Alive. 2018. https://www.hbw.com. Accessed Sep 2018.

[50] Francillon-Vieillot H, de Buffrénil V, Castanet JD, Géraudie J, Meunier FJ, Sire JY, Zylberberg L, de Ricqlès A, Carter JG (1990). Microstructure and mineralization of vertebrate skeletal tissues. Skeletal biomineralization: patterns, processes and evolutionary trends.

[51] O'connor PM (2009). Evolution of archosaurian body plans: skeletal adaptations of an air-sac-based breathing apparatus in birds and other archosaurs. J Exp Zool A Ecol Genet Physiol.

[52] Prum RO, Berv JS, Dornburg A, Field DJ, Townsend JP, Lemmon EM (2015). Lemmon AR. A comprehensive phylogeny of birds (Aves) using targeted next-generation DNA sequencing. Nature.

[53] Zondek B (1936). Impairment of anterior pituitary functions by follicular hormone. Lancet.

[54] Pfeiffer CA, Gardner WU (1938). Skeletal changes and blood serum calcium level in pigeons receiving estrogens. Endocrinology..

[55] Gardner WU, Pfeiffer CA (1943). Influence of estrogens and androgens on the skeletal system. Physiol Rev.

[56] Bowman BM, Miller SC (1986). The proliferation and differentiation of the bone-lining cell in estrogen-induced osteogenesis. Bone..

[57] Crisp E (1857). On the presence or absence of air in the bones of birds. Proc Zool Soc Lond.

[58] Cubo J, Casinos A (2000). Incidence and mechanical significance of pneumatization in the long bones of birds. Zool J Linnean Soc.

[59] Gutzwiller SC. Postcranial skeletal pneumaticity, bone structure, and foraging style in two clades of neognath birds. Unpublished BS thesis: Ohio University; 2010.

[60] Smith ND (2012). Body mass and foraging ecology predict evolutionary patterns of skeletal pneumaticity in the diverse “waterbird” clade. Evolution..

[61] O'Connor PM, Claessens LP (2005). Basic avian pulmonary design and flow-through ventilation in non-avian theropod dinosaurs. Nature.

[62] Brusatte SL, O’Connor JK, Jarvis ED (2015). The origin and diversification of birds. Curr Biol.

[63] Bellairs ADA, Jenkin CR, Marshall AJ (1960). The skeleton of birds. Biology and comparative physiology of birds.

[64] Hogg DA (1984). The development of pneumatisation in the postcranial skeleton of the domestic fowl. J Anat.

[65] Schepelmann K (1990). Erythropoietic bone marrow in the pigeon: development of its distribution and volume during growth and pneumatization of bones. J Morphol.

[66] Campana A (1875). Anatomie de l'appareil pneumatique-pulmonaire, etc., chez le poulet.

[67] Bremer JL (1940). The pneumatization of the humerus in the common fowl and the associated activity of theelin. Anat Rec.

[68] Lentacker AN, Van Neer WIM (1996). Bird remains from two sites on the Red Sea coast and some observations on medullary bone. Int J Osteoarchaeol.

[69] Hogg DA (1984). The distribution of pneumatisation in the skeleton of the adult domestic fowl. J Anat.

[70] King AS (1957). The aerated bones of Gallus domesticus. Cells Tissues Organs.

[71] Gutzwiller SC, Su A, O'Connor PM (2013). Postcranial pneumaticity and bone structure in two clades of neognath birds. Anat Rec.

[72] Müller B (1908). The air-sacs of the pigeon. Smithson Misc Collect.

[73] Apostolaki NE, Rayfield EJ, Barrett PM (2015). Osteological and soft-tissue evidence for pneumatization in the cervical column of the ostrich (*Struthio camelus*) and observations on the vertebral columns of non-volant, semi-volant and semi-aquatic birds. PLoS One.

[74] Harrison JG (1958). Skull pneumaticity-skull pneumaticity in wildfowl in relation to their mode of life. Wildfowl..

[75] Ekarius C. Storey’s illustrated guide to poultry breeds. North Adams: Storey publishing; 2007.

[76] Chinsamy A, Tumarkin-Deratzian A (2009). Pathologic bone tissues in a Turkey vulture and a nonavian dinosaur: implications for interpreting endosteal bone and radial fibrolamellar bone in fossil dinosaurs. Anat Rec.

[77] Benson RB, Butler RJ, Carrano MT, O'connor PM (2012). Air-filled postcranial bones in theropod dinosaurs: physiological implications and the ‘reptile’–bird transition. Biol Rev.

[78] Wedel MJ (2003). Vertebral pneumaticity, air sacs, and the physiology of sauropod dinosaurs. Paleobiology..

[79] Wedel MJ (2006). Origin of postcranial skeletal pneumaticity in dinosaurs. Integr Zool.

[80] Janensch W. Pneumatizitat bei Wirbeln von Sauropoden und anderen Saurischien. Palaeontographica. 1947:1–25.

[81] Schwarz D, Fritsch G (2006). Pneumatic structures in the cervical vertebrae of the late Jurassic Tendaguru sauropods *Brachiosaurus brancai* and *Dicraeosaurus*. Eclogae Geol Helv.

[82] Wedel MJ (2009). Evidence for bird-like air sacs in saurischian dinosaurs. J Exp Zool.

[83] Butler RJ, Barrett PM, Gower DJ (2009). Postcranial skeletal pneumaticity and air-sacs in the earliest pterosaurs. Biol Lett.

[84] Sereno PC, Wilson JA, Larsson HC, Dutheil DB, Sues HD (1994). Early cretaceous dinosaurs from the Sahara. Science.

[85] Cerda IA, Salgado L, Powell JE (2012). Extreme postcranial pneumaticity in sauropod dinosaurs from South America. Paläont Z.

[86] Claessens LP, O'Connor PM, Unwin DM (2009). Respiratory evolution facilitated the origin of pterosaur flight and aerial gigantism. PLoS One.

[87] Lambertz M, Bertozzo F, Sander PM (2018). Bone histological correlates for air sacs and their implications for understanding the origin of the dinosaurian respiratory system. Biol Lett.

[88] Simkiss K (1961). Calcium metabolism and avian reproduction. Biol Rev.

[89] Canoville A, Schweitzer MH, Zanno LE (2018) Data from: Systemic distribution of medullary bone in the avian skeleton: ground truthing criteria for the identification of reproductive tissues in extinct Avemetatarsalia. MorphoSource repository, project # P640. https://www.morphosource.org/Detail/ProjectDetail/Show/project_id/640. 10.1186/s12862-019-1402-7 PMC6407237 30845911

[90] Lamm ET, Padian K, Lamm ET (2013). Preparation and sectioning of specimens. Bone histology of fossil tetrapods: advancing methods, analysis, and interpretation.

