# Supplementary figures and images for: Systemic distribution of medullary bone in the avian skeleton: ground truthing criteria for the identification of reproductive tissues in extinct Avemetatarsalia
Source: BMC Evol Biol. 2019 Mar 7;19:71. doi: 10.1186/s12862-019-1402-7 (PMC6407237; doi:10.1186/s12862-019-1402-7)

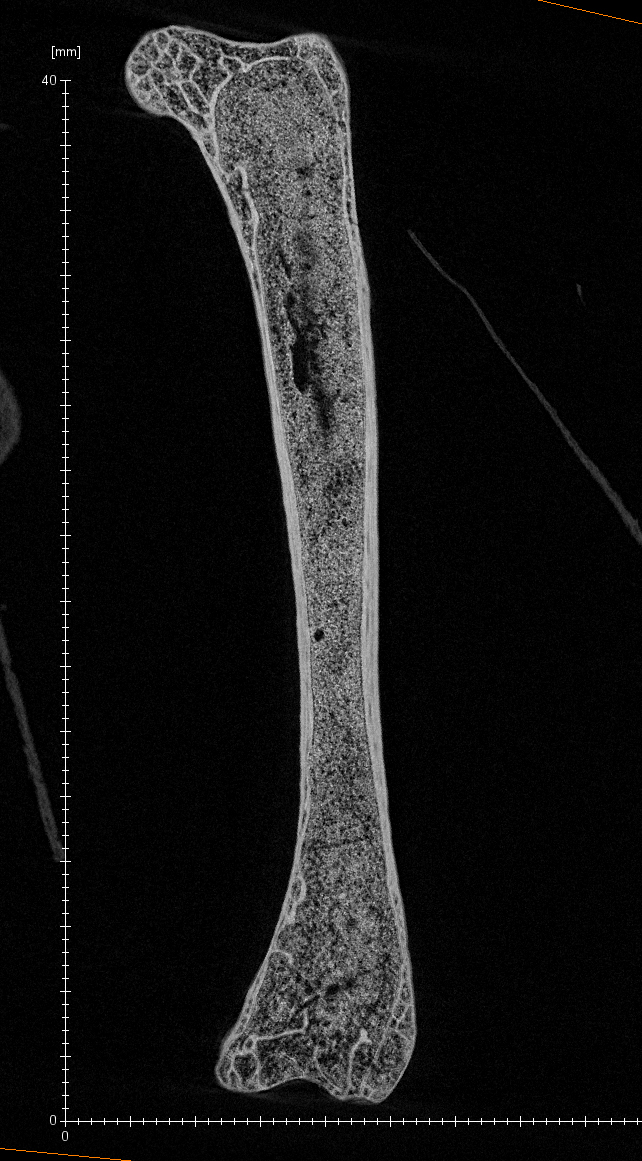

Supplement: Supplementary file 3 — Figure S1. Virtual longitudinal section of the femur of Phaethon rubricauda (USNM-631988). MB fills up most of the medullary cavity, as well as inter-trabecular spaces in both epiphyses. (JPG 486 kb) [file 12862_2019_1402_MOESM3_ESM.jpg]

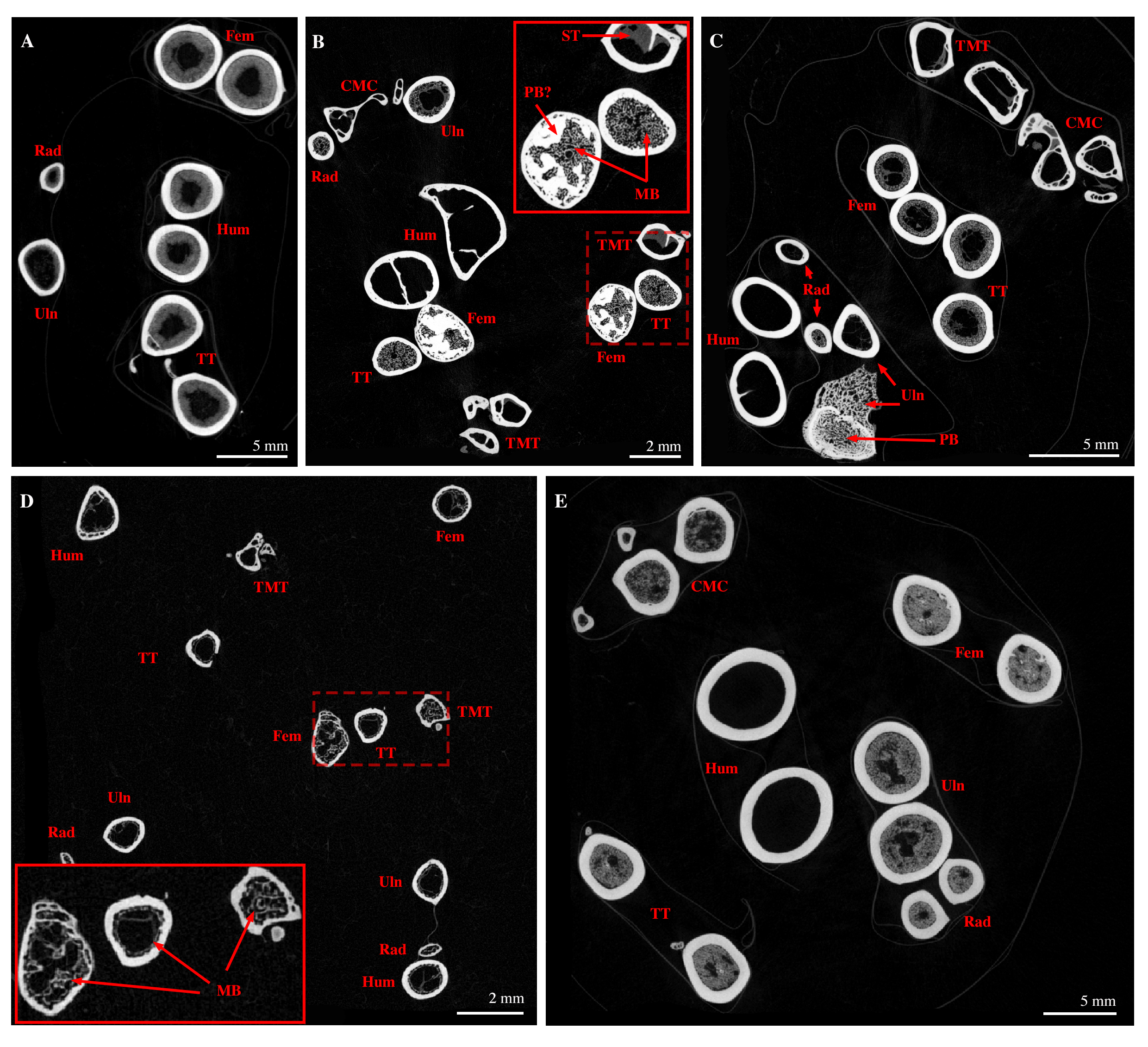

Supplement: Supplementary file 6 — Figure S2. Virtual cross-sections of limb bones in four different bird species. A: Limnodromus griseus (TMM-M8889). MB is present in the medullary cavities of all figured skeletal elements, including the humeri; B: Ptilinopus melanospilus (TMM-M11422). In this specimen, MB is not present in the humeri, carpometacarpi, and tarsometatarsi. As in all other specimens, MB has a granular aspect, while soft tissues appear smooth. Both femora show an unusual and very dense endosteal bone tissue (probably pathological) that fills up part of the medullary cavity, but is easily distinguishable from the neighboring MB; C: Colinus virginianus (TMM-M6536). In this specimen, MB is not present in the humeri, carpometacarpi, and tarsometatarsi. One ulna presents a pathology, with a periosteal pathological bone tissue and an endosteal pathological bone tissue that is clearly distinguishable from MB, based on mineralization rate and microstructure; D: Lonchura punctulata (TMM-M6977). MB is present in the medullary cavities of all figured skeletal elements, including the humeri and tarsometatarsi, although in very small quantities. E: Anas luzonica (TMM-M5395). MB is not present in the humeri of this specimen, but is clearly visible in the carpometacarpi. Abbreviations: CMC, carpometacarpus; Fem, femur; Hum, humerus; MB, medullary bone; PB, pathological bone; Rad, radius; ST, soft tissue; TT, tibiotarsus; TMT, tarsometatarsus; Uln, ulna. (JPG 2509 kb) [file 12862_2019_1402_MOESM6_ESM.jpg]
